# Enhanced Expression of *IL32* mRNA in Skeletal Muscles in the Context of Head and Neck Carcinomas

**DOI:** 10.1002/jcsm.70160

**Published:** 2025-12-28

**Authors:** Imane Baïche, Héla Hachicha, Thierry Ragot, Céline Gracia, Aurore Gelin, Anaïs Gader, Caroline Even, Ingrid Breuskin, Odile Casiraghi, Thibault Dayris, Filippo Dall'Ollio, Catherine Brenner, Karim Benihoud, Yegor Vassetzky, François Bidault, Philippe Gorphe, Pierre Busson, Fei Chen

**Affiliations:** ^1^ CNRS UMR 9018‐METSY Gustave Roussy and Université Paris‐Saclay Villejuif France; ^2^ Service de Cancérologie Cervico‐Faciale Gustave Roussy and Université Paris‐Saclay Villejuif France; ^3^ Département de Biologie et de Pathologie Médicales Gustave Roussy and Université Paris‐Saclay Villejuif France; ^4^ Bioinformatics Platform Gustave Roussy and Université Paris‐Saclay Villejuif France; ^5^ Koltzov Institute of Developmental Biology Moscow Russia; ^6^ Service d'Imagerie Diagnostique, Gustave Roussy and Université Paris‐Saclay Villejuif France

**Keywords:** fresh muscle fragment, head and neck carcinoma, human immortalised myoblast, IL32, sarcopenia

## Abstract

**Background:**

Cancer‐related sarcopenia (CRS) is a significant complication of head and neck carcinoma (HNC), characterised by muscle degeneration and poor clinical outcomes. Although various dietary and therapeutic interventions have been explored, most of them remain empirical, and the molecular mechanisms underlying CRS are not yet fully understood.

**Methods:**

Transcription profiles of muscle fragments from 29 HNC patients and 8 control donors were analysed by bulk RNA sequencing (6/29 and 3/8) and/or RT‐qPCR (29/29 and 5/8). In parallel, differentiating human myoblasts (AB1190) were subjected to indirect co‐culture with two types of effector cells: HNC cells (FaDu) or control epithelial cells (NHEK). The contactless effects of effector cells on target myoblasts were investigated using cell imaging to assess muscular differentiation, RT‐qPCR and Western blot to assess gene expression.

**Results:**

Bulk RNA sequencing identified 789 differentially expressed transcripts between HNC and control samples. Subsequent RT‐qPCR analysis focused on *IL32* and *BIRC3* mRNAs (up‐regulated in HNC samples) and *ACE1* mRNA (down‐regulated). Among male HNC patients, the *IL32/ACE1* mRNA ratio was significantly elevated in CRS cases (*p* = 0.0001, effect size *r* = 0.57) and correlated with the severity of muscle atrophy (negative correlation with the Skeletal Muscle Index at a threshold of 10%: *p* = 0.093, *r* = −0.41). In contrast, no such trend was observed for the *BIRC3/ACE1* ratio. Exposure of human myoblasts to HNC cells induced inhibition of myogenesis and strong up‐regulation of IL32 mRNA and protein. In contrast, these effects were absent or much smaller under exposure to NHEK controls.

**Conclusions:**

IL32 is a potential biomarker for CRS in HNC patients. In addition, the HNC–myoblast co‐cultivation model provides a promising in vitro system to study CRS mechanisms, potentially reducing the reliance on animal models.

## 1 Introduction

Carcinomas of the upper aero‐digestive tract or ‘head and neck carcinomas’ (HNCs) are epithelial tumours derived from the mucosae of the oral cavity, oropharynx, hypopharynx, larynx, paranasal sinuses, nasopharynx and nasal cavity. Despite their anatomic diversity, HNCs often share similar histological features. More than 90% of them are squamous cell carcinomas. HNCs represent the seventh leading cause of cancer in the world with an incidence of 890,000 new cases per year [1]. In most cases, the main etiological factors are alcohol and tobacco abuse. A substantial fraction of HNCs also results from chronic infection of the pharyngeal mucosa by oncogenic subtypes of the human papillomavirus [2]. Surgery and/or concomitant chemo‐radiotherapy are the standard of care for most HNCs regardless of their aetiology. Despite aggressive treatment, more than 50 % of them will relapse in the next 2 years.

Cachexia, a multifactorial syndrome combining chronic fatigue, weight loss and poor appetite, is highly prevalent in HNCs. One major component of cachexia is cancer‐related sarcopenia (CRS) characterised by a loss of skeletal muscle mass (with or without a concomitant loss of fat) that cannot be fully reversed by conventional nutritional support [3, 4]. CRS is characterised by a negative protein and energy balance due to a combination of reduced food intake and abnormal metabolism. Many tumours remotely cause a reprogramming of metabolism and cell fate in distant organs and tissues, especially in skeletal muscles [3, 4]. The mediators of these remote actions include hormones, cytokines, metabolites, proteins and microRNAs [5, 6, 7]. In current clinical practice, the diagnosis of cancer cachexia is usually based on clinical parameters: weight loss > 5% over the past 6 or 12 months and/or body mass index < 20 kg/m^2^ [8]. The diagnosis of CRS is often based on anthropometric measurements, for example, measurements of the skeletal muscle area at L3 on CT scans [9].

The overall prevalence of CRS in HNCs is probably higher than 50%, although there is no consensus on this disorder due to the absence of common assessment methods [10] [S1, S2]. CRS is a major factor predicting a poor outcome. Not only does it have a profound impact on the quality of life, but it is also associated with greater treatment toxicity and poorer prognosis [10]. Therefore, besides specific anti‐tumour treatments, many types of therapeutic interventions are currently being explored for a direct curative effect on skeletal muscle alterations. They include various dietary supplements, physical exercise under medical supervision and electro‐myo‐stimulation [11, 12]. There is a striking contrast between the multiplicity of these approaches and our poor knowledge of the cellular and molecular alterations underlying muscle wasting in situ. Most of our knowledge is based on extrapolations from animal models [4, 13, 14, 15]. Some data were obtained directly from muscle specimens mainly in the context of digestive tumours (intraoperative collection of fragments of the rectus abdominis muscle) but not in the context of HNCs [14, 16, 17]. This is not surprising since muscle biopsies are not a part of the initial standard assessment of most human malignancies including HNCs. Here, we took a first step towards the direct study of molecular and cellular alterations of muscles in the context of HNCs. We designed a pilot study based on muscle fragments taken intraoperatively from patients undergoing surgery for HNCs. The collection of these fragments was organised in connection with a surgical clinical trial designated ‘Magnolia’ aiming at a better definition of surgical margins using fluorescent imaging (NCT04842162). Besides these cancer‐related muscle fragments, control muscle fragments were obtained from donors without known malignancies or muscular diseases. Comparative bulk RNAseq revealed distinct transcription profiles for these two types of samples. One of the most consistent observations was a greater abundance of *IL32* mRNA in muscle fragments from HNC patients as compared to healthy controls. These findings were corroborated by in vitro experiments where the expression of both IL32 mRNA and protein was strongly enhanced in human myoblasts co‐cultured with malignant HNC cells, but not, or with a small amplitude, under exposure to non‐malignant human keratinocytes. Overall, our data are in favour of a humoural contribution of malignant cells to aberrant muscle alterations observed in the context of HNCs. One remarkable aspect of these alterations is an increase in the expression of IL32 mRNA and protein.

## 2 Materials and Methods

### 2.1 Muscle Fragments From Tumour‐Bearing Patients and Control Donors

Clinical samples used in this study were snap‐frozen fragments of skeletal muscles. The main information about these samples is summarised in Table 1. The collection of small muscle fragments (50 mm^3^) from tumour‐bearing patients was made intraoperatively in the frame of an ancillary study of the Magnolia clinical trial, with the approbation of the Ethics committee ‘Sud‐Est V’ given on February 13, 2020 (Magnolia trial: NCT4842162 and EudraCT 2019‐002442‐20). The inclusion and exclusion criteria are summarised in Table S1. Regarding fresh muscle pieces, the aim of the ancillary study was to investigate the biological characteristics of the muscle tissues in HNC patients. Most muscle pieces were cut from the sternocleidomastoid muscle during the exploration of cervical lymph nodes. In a few cases, they were cut from surgical tumour pieces or surgical flaps prepared for reconstructive surgery (haemostasis was performed after biopsies). Immediately after excision in the surgery room, the muscle pieces were minced into several fragments that were snap‐frozen except for one of them fixed in PBS‐paraformaldehyde 4% and paraffin‐embedded to check their muscular origin (Figure S1). All patients included in Magnolia were HNC patients undergoing initial surgical treatment at the Department of Head and Neck Surgery of Gustave Roussy.

**TABLE 1:** Clinical and demographic characteristics of the human subjects involved in the study.

Subject	Sex	Age	Muscle type	SMI^a^	Cancer‐related sarcopenia^b^	Tumour type	TNM stage
Ctrl A	F	60	Tensor fascia lata	—	—	—	—
Ctrl B	M	63	Tensor fascia lata	—	—	—	—
Ctrl C	F	59	Tensor fascia lata	—	—	—	—
Ctrl 1	M	60	Tensor fascia lata	—	—	—	—
Ctrl 2	M	55	Tensor fascia lata	—	—	—	—
Ctrl 3	F	55	Tensor fascia lata	—	—	—	—
Ctrl 4	M	47	Tensor fascia lata	—	—	—	—
Ctrl 5	M	72	Tensor fascia lata	—	—	—	—
Ctrl P1	F	60	—	—	—	—	—
Ctrl P2	M	63	—	—	—	—	—
Ctrl P3	F	59	—	—	—	—	—
Ctrl P4	M	60	—	—	—	—	—
Ctrl P5	M	55	—	—	—	—	—
Ctrl P6	F	55	—	—	—	—	—
HNC1	F	88	Sternocleidomastoid	29.0	Yes	Oral cavity	T4aN0M0
HNC2	M	53	Pectoralis major	45.3	Yes	Pharyngo‐larynx	T4aN2bM0
			Sternocleidomastoid				
HNC3	M	66	Sternocleidomastoid	35.6	Yes	Oral cavity	T4aN2bM0
			Pectoralis major				
HNC4	M	76	Sternocleidomastoid	43.5	Yes	Oral cavity	T3N0M0
			Quadriceps femoris				
HNC5	F	72	Latissimus dorsi	39.7	No	Maxillary sinus	T4aN2bM0
HNC6	M	63	Digastric	49.5	Yes	Oral cavity	T4aN0M0
HNC7	M	75	Masseter	51.2	Yes	Oral cavity	T4aN0M0
HNC8	M	57	Sternocleidomastoid	56.6	No	Oral cavity	T3N2cM0
HNC9	M	65	Sternocleidomastoid	40.7	Yes	Oral cavity	T4aN2bM0
HNC10	M	63	Serratus major	33.9	Yes	Oral cavity	T4aN0M0
HNC11	F	63	Quadriceps femoris	38.4	Yes	Oral cavity	T3N0M0
HNC12	M	62	Sternocleidomastoid	45.3	Yes	Oral cavity	T4aN3bM0
HNC13	M	70	Sternocleidomastoid	47.5	Yes	Oral cavity	T1N0M0
HNC14	F	81	Tongue	34.6	Yes	Oral cavity	T1N1M0
HNC15	M	54	Sternocleidomastoid	49.0	Yes	Oral cavity	T1N0M0
HNC16	F	57	Sternocleidomastoid	35.5	Yes	Oral cavity	T1N2M0
HNC17	M	23	Quadriceps femoris	68.9	No	Oral cavity	T2N1M0
HNC18	M	55	Sternocleidomastoid	59.9	No	Oral cavity	T2N0M0
HNC19	F	71	Sternocleidomastoid	33.5	Yes	Oral cavity	T2N2M0
HNC20	F	64	Sternocleidomastoid	55.4	No	Oral cavity	T2N1M0
HNC21	M	47	Sternocleidomastoid	50.2	Yes	Oral cavity	T2N0M0
HNC22	M	46	Sternocleidomastoid	58.8	No	Oropharynx	T1N0M0
HNC23	M	68	Quadriceps femoris	45.2	Yes	Oral cavity	T3N2aM0
HNC24	M	60	Sternocleidomastoid	56.9	No	Oral cavity	T2N3bM0
HNC25	F	57	Sternocleidomastoid	36.9	Yes	Oral cavity	T1N0M0
HNC26	M	68	Sternocleidomastoid	42.8	Yes	Oral cavity	T3N2M0
HNC27	M	72	Sternocleidomastoid	43.7	Yes	Oral cavity	T3N0M0
HNC28	M	63	Sternocleidomastoid	57.9	No	Oral cavity	T2N0M0
HNC29	M	61	Sternocleidomastoid	47.1	Yes	Oral cavity	T3N2bM0

^a^ At L3 (based on C3 CT scan).

^b^ As explained in the Materials and Methods section, the threshold for cancer‐related sarcopenia was set at 52.4 and 38.5 cm^2^/m^2^ for male and female patients, respectively.

Control skeletal muscle fragments were provided by Myobank‐AFM (Myology Institute, Paris). They were initially obtained from anonymous volunteers undergoing hip surgery and free of malignancies or muscular diseases.

Overall, these clinical samples were processed in three series with partial overlaps (Tables 1 and 2 and Table S2). The first series was used for bulk RNAseq and named the HNC‐RNAseq series (Table S2). It included nine HNC‐related muscle fragments taken from six patients (HNC1 to 6), four men (mean age 64.5) and two women (mean age 80). These HNC‐related samples were matched with three control fragments (Ctrl A to C) from one man (aged 63) and two women (mean age 59.5). The second series was used for PCR analysis and named the HNC‐PCR series. It included 29 tumour‐related muscle fragments (HNC1 to 29) from 21 men (mean age 60.3) and 8 women (mean age 69.1) matched to five control fragments (Ctrl 1 to 5) from four men (mean age 58.5) and one woman (aged 55) (Table 2).

**TABLE 2:** PCR analyses in connection with some demographic and clinical characteristics (HNC‐PCR series and corresponding control).

Subject	Sex	Age	Muscle type	SMI^**a**^	Cancer‐related sarcopenia^**b**^	IL32/PPIA	BIRC3/PPIA	ACE1/PPIA	IL32/ACE1	BIRC3/ACE1
						2^‐ (Ct IL32 ‐ Ct PPIA)^	2^‐ (Ct BIRC3 ‐ Ct PPIA)^	2 ^‐ (Ct ACE1 ‐ Ct PPIA)^	2^‐ (Ct IL32 ‐ Ct ACE1)^	2^‐ (Ct BIRC3 ‐ Ct ACE1)^
Ctrl 1	M	60	Tensor fascia lata	—	—	0.265	0.008	0.014	18.290	0.570
Ctrl 2	M	55	Tensor fascia lata	—	—	0.322	0.001	0.013	24.489	0.096
Ctrl 3	F	55	Tensor fascia lata	—	—	0.991	0.005	0.024	41.247	0.221
Ctrl 4	M	47	Tensor fascia lata	—	—	0.225	0.003	0.015	15.399	0.225
Ctrl 5	M	72	Tensor fascia lata	—	—	0.261	0.003	0.012	21.308	0.209
HNC1	F	88	Sternocleidomastoid	29.0	Yes	3.135	0.021	0.004	815.506	5.334
HNC2	M	53	Sternocleidomastoid	45.3	Yes	13.470	0.040	0.006	2399.655	7.108
HNC3	M	66	Sternocleidomastoid	35.6	Yes	1.896	0.017	0.001	1380.706	12.047
HNC4	M	76	Sternocleidomastoid	43.5	Yes	4.063	0.017	0.005	776.973	3.268
HNC5	F	72	Latissimus dorsi	39.7	No	1.049	0.019	0.010	107.262	1.917
HNC6	M	63	Digastric	49.5	Yes	2.221	0.015	0.004	600.035	4.020
HNC7	M	75	Masseter	51.2	Yes	1.328	0.011	0.016	82.705	0.665
HNC8	M	57	Sternocleidomastoid	56.6	No	0.809	0.022	0.005	157.923	4.316
HNC9	M	65	Sternocleidomastoid	40.7	Yes	1.213	0.034	0.003	386.222	10.815
HNC10	M	63	Serratus major	33.9	Yes	8.615	0.012	0.006	1625.272	2.240
HNC11	F	63	Quadriceps femoris	38.4	Yes	4.810	0.026	0.018	262.925	1.391
HNC12	M	62	Sternocleidomastoid	45.3	Yes	5.718	0.032	0.002	3125.220	17.374
HNC13	M	70	Sternocleidomastoid	47.5	Yes	6.246	0.049	0.011	600.164	4.719
HNC14	F	81	Tongue	34.6	Yes	0.452	0.012	0.008	57.644	1.528
HNC15	M	54	Sternocleidomastoid	49.0	Yes	9.519	0.006	0.008	1145.692	0.664
HNC16	F	57	Sternocleidomastoid	35.5	Yes	0.143	0.016	0.006	23.673	2.675
HNC17	M	23	Quadriceps femoris	69.0	No	2.903	0.016	0.007	434.519	2.337
HNC18	M	55	Sternocleidomastoid	59.9	No	0.977	0.005	0.004	264.795	1.283
HNC19	F	71	Sternocleidomastoid	33.5	Yes	3.419	0.011	0.013	278.042	0.910
HNC20	F	64	Sternocleidomastoid	55.4	No	1.507	0.033	0.004	335.189	7.095
HNC21	M	47	Sternocleidomastoid	50.2	Yes	4.485	0.019	0.005	1014.594	4.370
HNC22	M	46	Sternocleidomastoid	58.8	No	3.176	0.006	0.006	565.915	1.144
HNC23	M	68	Quadriceps femoris	45.2	Yes	1.378	0.016	0.010	149.695	1.697
HNC24	M	60	Sternocleidomastoid	56.9	No	0.407	0.052	0.010	39.531	4.978
HNC25	F	57	Sternocleidomastoid	36.9	Yes	1.996	0.096	0.006	362.947	17.561
HNC26	M	68	Sternocleidomastoid	42.8	Yes	6.819	0.017	0.010	696.122	1.756
HNC27	M	72	Sternocleidomastoid	43.7	Yes	1.843	0.007	0.006	334.092	1.315
HNC28	M	63	Sternocleidomastoid	57.9	No	8.189	0.007	0.003	3111.555	2.837
HNC29	M	61	Sternocleidomastoid	47.1	Yes	9.356	0.006	0.003	3156.795	1.868

^a^ At L3 (based on C3 CT scan).

^b^ As explained in the Materials and Methods section, the threshold for cancer‐related sarcopenia was set at 52.4 and 38.5 cm^2^/m^2^ for male and female patients, respectively.

### 2.2 Skeletal Muscle Index Measurement

The assessment of the skeletal muscle mass was based on computed tomography (CT) scans made prior to any treatment, either extended to whole body (15 patients) or restricted to head and neck (14 patients). Muscle areas at C3 level were delineated on CT images using the Horos software (Version 4.0.0), an open‐source medical imaging platform designed for macOS (https://github.com/horosproject/horos/releases, accessed on 29 October 2023). Each patient's muscle area was outlined three times independently to ensure precision, with the final cross‐sectional area (CSA) value taken as the average of these measurements. Based on the algorithm given by Swartz et al. [18], the CSA at L3 was extrapolated using the formula: CSA at L3 (cm^2^) = 27.304 + 1.363 × CSA at C3 (cm^2^) – 0.671 × age (y) + 0.640 × weight (kg) + 26.442 × gender (male = 2, female = 1). For 15 patients, it was possible to assess the paravertebral muscle area at the levels of both L3 and C3. As shown in Figure S2, for the 15 patients with whole‐body CT scan, there was a very good correlation between skeletal muscle indices (SMIs) deduced from direct measurements at L3 or extrapolations from C3 measurements.

Finally, the SMI was calculated by normalising the skeletal muscle area according to the subject height (SMI = CSA of skeletal muscle at L3/Height squared). The cut‐off values for muscular atrophy defined by Fearon et al. were 52.4 cm^2^/m^2^ for males and 38.5 cm^2^/m^2^ for females [19].

### 2.3 Total RNA Extraction

Total RNA extraction from muscle fragments (30 mg of tissue) was performed using the RNA‐Easy Fibrous Tissue Kit (Qiagen). The first step was tissue homogenisation in the lysis buffer using the Tissue Ruptor grinder from Qiagen. For cultured myoblasts, total RNA was extracted using the RNeasy Mini Kit (Qiagen) including cell solubilisation by simple application of the lysis buffer.

### 2.4 Bulk RNA Sequencing and Bioinformatics Analysis

Bulk RNA sequencing was performed using 100 bp paired‐end reads (NovaSeq 6000, Illumina) with 60 million reads per sample. Raw data are available in the European Genome‐Phenome Archive (EGAD50000000944). Transcript abundance was quantified using Salmon (Gencode v27), and differential expression analysis was conducted using the DESeq2 R package. Genes with adjusted *p* < 0.05 and |log_2_ fold change| > 1 were considered significantly differentially expressed. To account for within‐patient correlation, we also applied the Limma + Voom pipeline with the duplicateCorrelation function [20], treating patient ID as a blocking factor. Additional technical details are provided in the Supplementary Materials and Methods.

### 2.5 Quantitative RT‐PCR Analysis

Total RNA was converted into cDNA using the Reverse Transcriptase Core kit (Eurogentec, RT‐RTCK‐03). The quantitative PCR was performed on a real‐time PCR detection system Step One Plus (Applied Biosystems) using the FastStart Universal SYBR Green Master (Rox) (Merck–Sigma–Aldrich), with five technical replicates for each pathological or experimental condition. The Q‐PCR program was as follows: 10 min at 95°C followed by 40 cycles: 15 s at 95°C, 60 s at 60°C and 60 s at 72°C. Primer pairs are described in Table S3. Relative gene expression was assessed using the 2^−ΔCq^ or 2^−ΔΔCq^ method, as appropriate. Detailed methodological information, including the normalisation strategy, is provided in the Supplementary Materials and Methods.

### 2.6 Cell Lines Propagated In Vitro

In vitro studies mainly consisted of indirect co‐cultures of immortalised myoblasts (AB1190) with epithelial cells, either malignant HNC cells (FaDu) or normal keratinocytes (NHEK). FaDu and AB1190 cells propagated in our lab were recently subjected to STR analysis (Table S4). FaDu cells were derived from a hypopharyngeal squamous cell carcinoma (Cellausorus Accession Number: CVCL‐1218) and have already been used in our laboratory [21]. AB1190 myoblasts have been kindly provided by Dr. Vincent Mouly (Institut de Myologie, Paris) [22]. Myoblast differentiation was induced by switching to DMEM culture medium with only 2% FCS as described elsewhere [23]. Details on culture and media compositions and additives are provided in Supplementary Materials and Methods.

### 2.7 Indirect Co‐Culture of Myoblasts and Carcinoma Cells

Indirect co‐cultures of AB1190 cells with epithelial cells were done using six‐well culture plates with internal cups (Transwell, Millicell, Merck, 0.4 μm). Internal cups and well bottoms delineate the upper and lower compartments, respectively. At Day 0, both target myoblasts and interfering epithelial cells were seeded in myoblast growth medium. Epithelial cells (FaDu or NHEK) were seeded in the upper compartment, in 2 mL of medium, in various amounts (from 15,000 to 120,000 cells/cup). Myoblasts (AB1190) were seeded in the lower compartments, in 3 mL of medium, at 1.2 × 10^5^ cells/well. Twenty‐four hours later (Day 1), the growth medium was replaced by the differentiation medium in both compartments. For the basic control condition, myoblasts were seeded in the absence of any cell in the upper compartment. At Day 5, after 96 h of incubation in the differentiation medium and prior to cell collection, AB1190 cells were stained with CellTracker (ref. C7025, Thermo Fisher) to visualize the cytoplasm and Hoechst 33342 to label the nuclei. Imaging was done using a Cytation1 system (Agilent).

### 2.8 Western Blot

At Day 5 of co‐culture, protein extracts were made from AB1190 myoblasts, separated on 4–20% Bis–Tris gels (mPAGE MP42G10, Millipore) and transferred onto PVDF membranes (Millipore). Membranes were incubated overnight at 4°C with primary antibodies against IL32 (ab315466, Abcam), myosin heavy chain (MHC—MAB4470, Bio‐Techne) and β‐actin (sc‐81178, Santa Cruz). Based on the peptide sequence used for immunisation, ab315466 is predicted to detect human IL32 Isoform 1 (gamma), Isoform 2 (beta), Isoform 3 (delta) and Isoform 4 (alpha). Secondary HRP‐conjugated antibodies were used to reveal bands. Band intensity was quantified using the ImageJ software, and target protein levels were normalised to the corresponding loading control (β‐actin).

### 2.9 Statistical Analysis

All statistical analyses were performed using Prism 9 (GraphPad Software, San Diego, CA, USA) and R software (Version 4.4.1). Prior to conducting group comparisons and correlation analysis, outliers were excluded using the interquartile range (IQR) method, which identifies values falling below the first quartile (Q1)—1.5 × IQR or above the third quartile (Q3) + 1.5 × IQR.

For comparisons between two groups, the Mann–Whitney *U* test was used. For multiple groups, normality and variance homogeneity were assessed; one‐way ANOVA was applied when assumptions were met; otherwise the Kruskal–Wallis test was used. Pearson or Spearman correlation was conducted depending on data distribution. Multivariate linear regression was performed in R to assess the independent effects of age (continuous), gender, tumour stage (T1–T2 vs. T3–T4) and sarcopenia (present vs. absent) on *IL32* mRNA expression.

All statistical tests were two‐tailed. A *p* < 0.05 was considered statistically significant except for multivariate linear regression analysis where the threshold was set at 0.10. Statistical significance is indicated as follows: *p* < 0.05 (*), *p* < 0.01 (**), *p* < 0.001 (***) and *p* < 0.0001 (****).

## 3 Results

### 3.1 Transcriptional Alterations Occurring in Skeletal Muscles in the Context of HNCs

Nine muscle fragments from six HNC patients were obtained prior to any treatment in the frame of an ancillary study of the Magnolia protocol; they are listed in Table 1 and Table S2 (HNC1 to 6) (HNC‐RNAseq series). Four of the donor patients were males (mean age 64.5) and two females (mean age 80). Five muscle fragments were collected in situ at the stage of lymph node dissection, from neck muscles, either from the sternocleidomastoid (SCM, four samples) or the digastric (one sample) muscles. The other four fragments were collected from muscle flaps intended for reconstructive surgery: pectoralis major (two samples), femoris quadriceps (one sample) and latissimus dorsi (one sample). Both types of fragments were available for three patients (HNC2, 3 and 4), whereas only one type was available for patients HNC1, 5 and 6. Besides samples from HNC patients, control muscle fragments were collected from the tensor fascia lata of three patients undergoing hip surgery in the absence of known malignancy or muscle disease (Ctrl A, B and C) including two women and one man aged, respectively, 59, 60 and 63.

**FIGURE 1:**
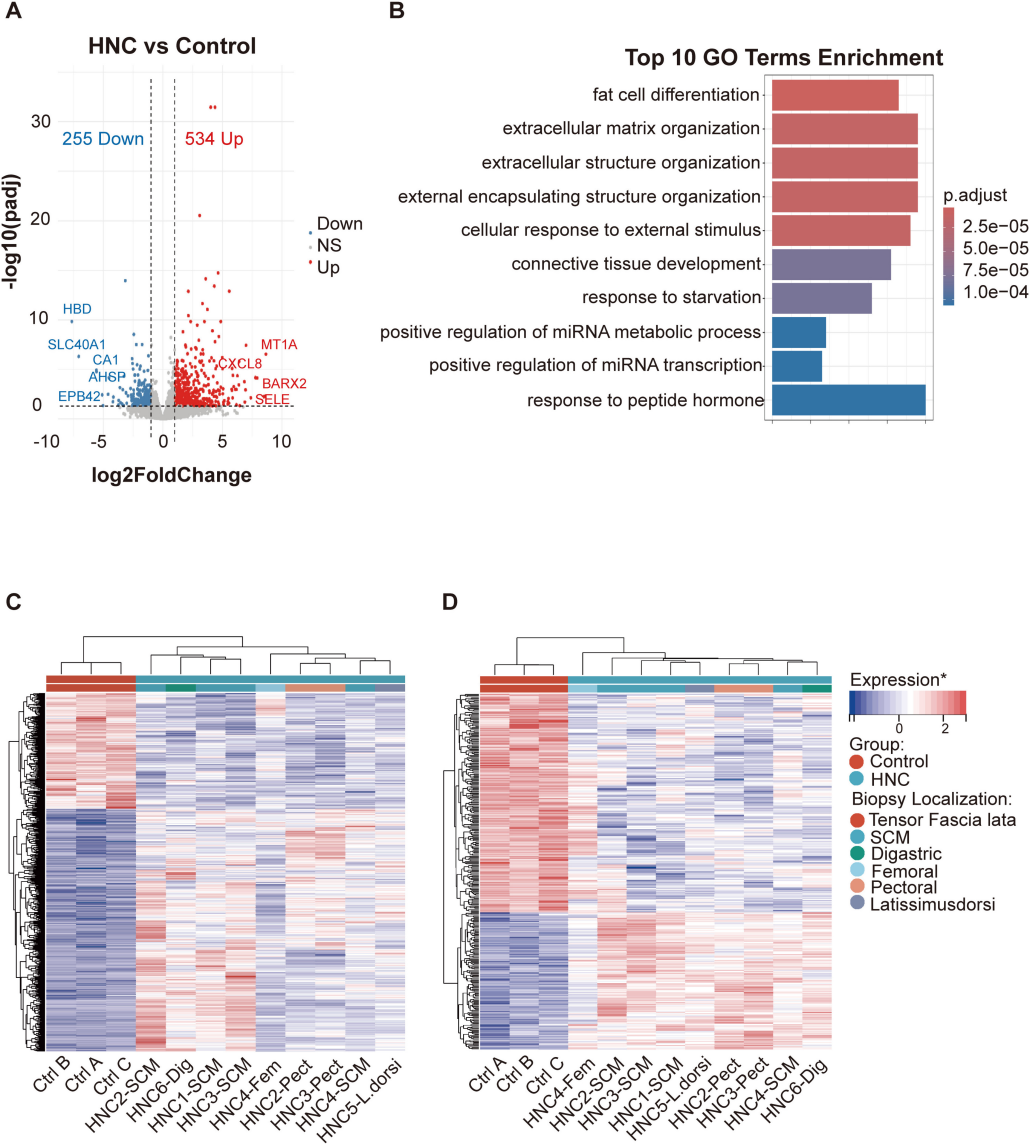
Comparative mRNA profiling of muscle samples from HNC patients and control donors. Nine muscle fragments from six HNC patients and three control fragments from three distinct donors were subjected to bulk RNAseq. HNC fragments had been collected either from cervical or flap muscles: sternocleidomastoid (SCM) (patients HNC1 to 4), pectoralis major (HNC2 and 3), quadriceps femoris (HNC4), latissimus dorsi (HNC5) and digastric muscle (HNC6) (Table S2). The control donors were volunteers undergoing hip surgery free of known muscular or malignant diseases. Their muscle fragments were taken from the tensor fascia lata. (A) Volcano plot showing differentially expressed genes (DEGs) between HNC patients and control donors: 789 of 19,216 genes with 534 up‐regulated and 255 down‐regulated genes in HNC muscle samples. (B) Top 10 enriched GO terms from the GO enrichment analysis. (C and D) Supervised heatmap and dendrogram taking into account the 789 DEGs and built using either the DESq2 (C) or the Limma + Voom (D) pipelines. *Expression values were based on rlog‐transformed counts and normalised by gene‐wise *Z* score (row‐wise). Both genes and samples were clustered using hierarchical clustering. In both cases, there is an obvious separation of the HNC and control transcription profiles.

The count table of comparative bulk RNAseq for HNC and control muscles is presented in Table S5. The results of bioinformatics analysis are summarised in the Volcano plot and Gene Ontology enrichment analysis charts of Figure 1A,B; among 789 differentially expressed mRNAs, 534 were up‐regulated and 255 were down‐regulated (out of 19,216 genes). The main cellular functional networks overrepresented in HNC samples compared to the healthy controls were ‘fat cell differentiation,’ ‘extracellular matrix organisation’ and ‘extracellular structure organisation.’ Next, we looked at clusters of samples determined by gene profiles using heatmaps and dendrograms. Despite some heterogeneity among the transcription profiles of HNC samples, we found an obvious separation between the patterns of HNC and control muscle fragments (see the dendrograms and heatmaps generated using the DESq2 pipeline in Figure 1C). One aspect of the heterogeneity of HNC muscle samples was the separation of samples collected from neck muscles (sternocleidomastoid or digastric) and those from muscular flaps. They formed two distinct branches in the dendrogram. This separation also applied to profiles of muscle samples collected from the same patient at two distinct anatomic locations (HNC2, 3 and 4). For patients HNC2 and 3, the profiles of the paired muscle samples were split in the branches corresponding to the neck and flap muscles, respectively. For patient 4, the profiles of paired samples were less distant but still not adjacent. Nevertheless, the most significant separation was between muscle fragments from HNC patients and control donors. This was confirmed when using one additional RNAseq pipeline distinct from DESeq2 and more oriented towards sample sets of small size with possible contribution of several samples from the same individuals (Limma + Voom analysis pipeline) (Figure 1D) [20]. Overall, these data provide compelling evidence that, in the context of HNC, consistent alterations affect the gene expression profile of skeletal muscles.

## 4 Altered Transcription of *BIRC3*, *IL32* and *ACE1* Genes in Skeletal Muscles in the Context of HNCs

**FIGURE 2:**
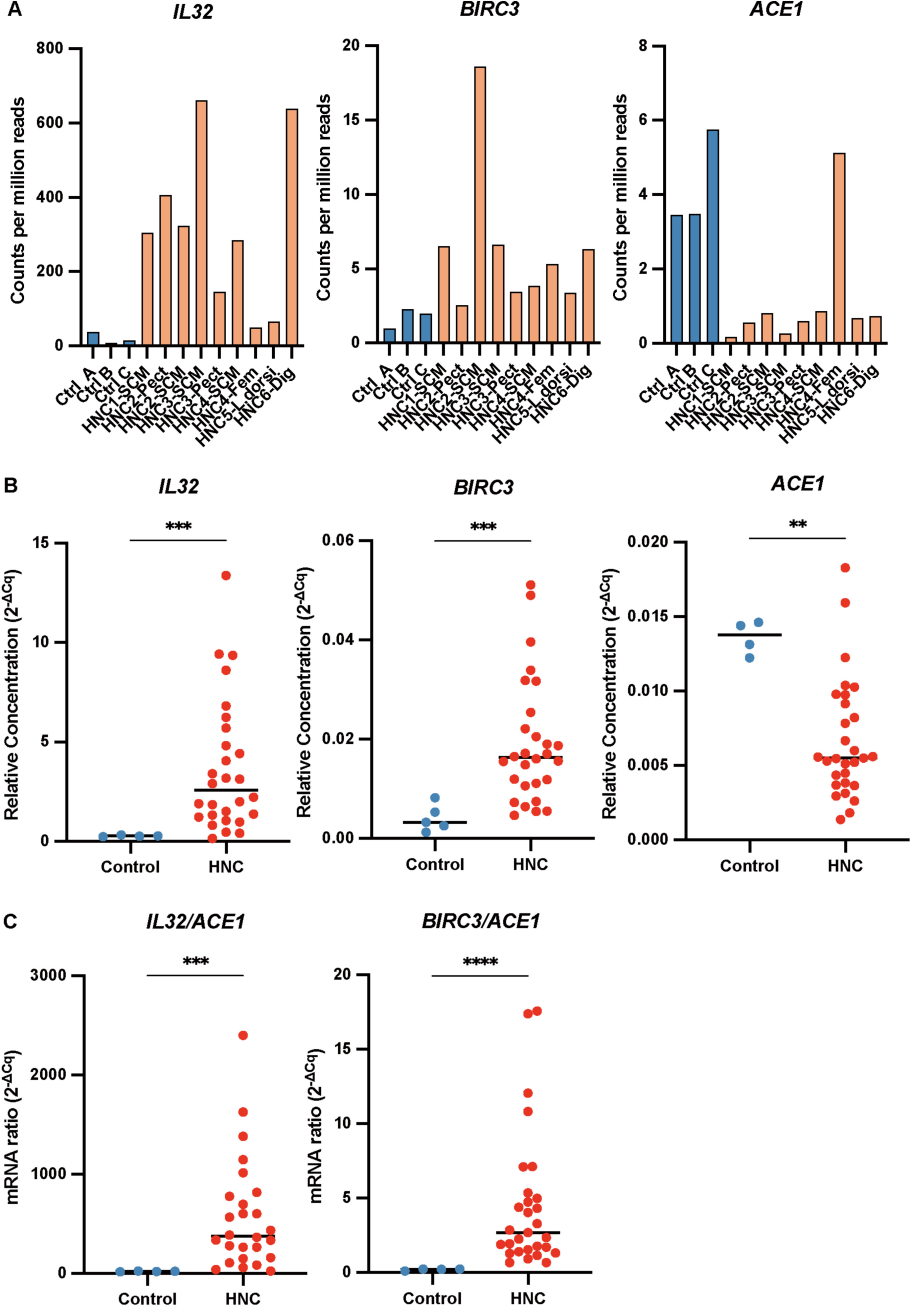
Altered transcription of *IL32*, *BIRC3* and *ACE1* mRNAs in skeletal muscles from HNC patients compared to control donors. (A) Evaluation by RNAseq of the abundances of *IL32*, *BIRC3* and *ACE1* mRNAs in the muscle samples described in the legend of Figure 1 (nine muscle fragments from six HNC donors vs. three control fragments). (B). The abundance of *IL32*, *BIRC3* and *ACE1* mRNAs was determined in muscle fragments from 29 HNC patients and five control donors (Table 2) by RT‐qPCR. Data were analysed using the 2^−ΔCq^ method. Internal calibrators were either *PPIA* (B) or *ACE1* (C) mRNAs. Statistical analysis was performed using the Mann–Whitney *U* test (median values are indicated by horizontal bars). Statistical significance is indicated as follows: *p* < 0.05 (*), *p* < 0.01 (**), *p* < 0.001 (***) and *p* < 0.0001 (****). *p* < 0.05 was considered statistically significant.

The next part of our study was focused on three genes differentially expressed in HNC and control muscles whose products, according to previous publications, are known to be involved in muscle pathology or HNC oncogenesis: *BIRC3*, *IL32* and *ACE1* [24, 25, 26] [S3]. *BIRC3* and *IL32* were selected among 534 genes up‐regulated in the HNC muscles of the RNAseq series vs. matched controls (see Figure 2A). Conversely, *ACE1* was selected among 255 genes down‐regulated in HNC muscles (Figure 2C). Each of these three genes is known to be expressed in physiological conditions in human skeletal muscles as shown by single‐cell RNAseq reported in the Protein Atlas database (accession Numbers: ENSG00000008517‐IL32/single + cell; ENSG00000023445‐BIRC3/single + cell; ENSG00000159640‐ACE/single + cell).

At the next step, the relative abundances of *IL32*, *BIRC3* and *ACE1* mRNAs in HNC vs. control samples were evaluated by quantitative RT‐qPCR applied on (1) samples from the extended HNC series (HNC1 to 29—HNC‐PCR series) and (2) a new control series (Ctrl 1 to 5) (Tables 1 and 2). The PCR HNC series included some muscle fragments previously used for bulk RNAseq. All these fragments were from the sternocleidomastoid except for HNC5 (latissimus dorsi). Samples of the ‘RNAseq series’ were combined with 23 additional fragments collected from 23 distinct donors. Overall, 21 muscle samples were collected from the sternocleidomastoid, three from other muscles of the Head and Neck area (one digastric, one masseter and one tongue) and five from flap muscles. Twenty‐one HNC patients were men (mean age 60.3) and eight women (mean age 69.1). Taking into account the ethical and practical difficulties of collecting these types of samples, we did our best to obtain gender and age distributions roughly similar to those of the PCR control series (more details available in the Materials and Methods section). As shown in Figure 2, the amounts of *BIRC3* and *IL32* transcripts were significantly greater in muscle fragments from HNC compared to healthy controls (*BIRC3*: *p* = 0.0004, effect size *r* = 0.55; *IL32*: *p* = 0.0007, effect size *r* = 0.52) (Figure 2B). In contrast, the amounts of *ACE1* were significantly lower in the HNC group (*p* = 0.0035, effect size *r* = 0.47) (Figure 2B). As expected, the differences between these two groups were even more striking when looking at the *IL32*/*ACE1* and *BIRC3*/*ACE1* mRNA ratios (*IL32/ACE1: p*  = 0.0001, effect size *r* = 0.57; BIRC3/ACE1: *p* < 0.0001, effect size *r* = 0.56) (Figure 2C). It should be noted that the expression levels of *BIRC3* and *ACE1* were significantly lower than those of *IL32* (Figure 2).

One limitation of the previous comparisons was that all muscle fragments from control donors were obtained from a unique type of skeletal muscle—tensor fascia lata—that was not represented in the panel of muscle fragments obtained from HNC patients. Therefore, one could not formally rule out that the observed differences in *IL32*, *BIRC3* and *ACE1* mRNA abundances reflected physiological characteristics linked to muscle anatomic location rather than pathological alterations resulting from the malignant context. Therefore, we analysed the expression of these three mRNAs in connection with the severity of clinical muscle alterations. When looking at the whole series of HNC patients, we found a greater amount of *IL32* mRNA in muscle samples obtained from sarcopenic compared to non‐sarcopenic patients (*p* = 0.0233, effect size *r* = 0.43) (Table 2 and Figure S3A). The concentration of *IL32* mRNAs was also influenced by patient gender (*p* = 0.0195, effect size *r* = 0.86) (Figure S4). However, multivariate analysis taking into account patient age and gender as well as primary tumour size was in favour of an independent role of the skeletal muscle status (sarcopenic or not sarcopenic). Detailed results are provided in Supplementary File S1.

**FIGURE 3:**
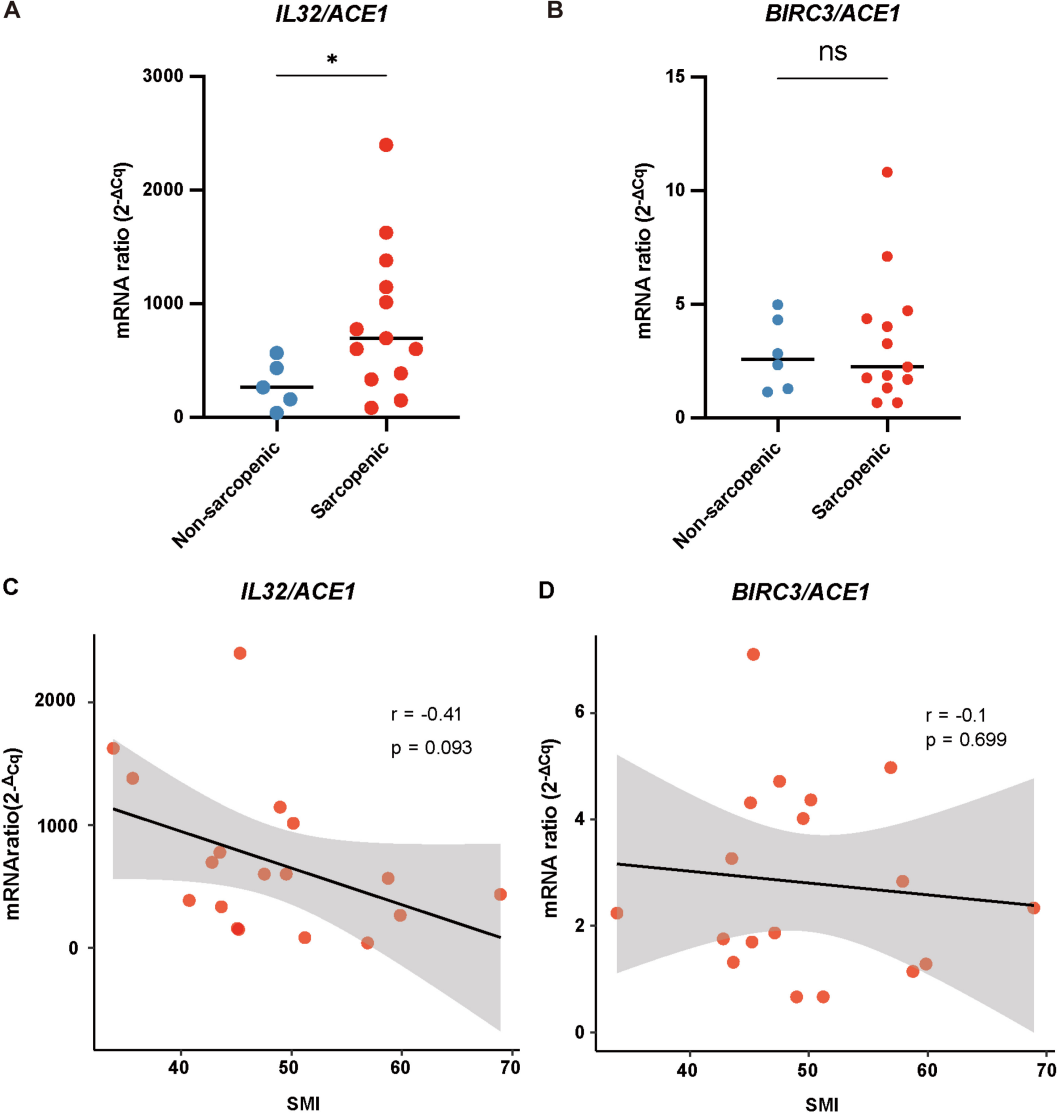
IL32/ACE1 and BIRC3/ACE1 mRNA ratios in muscle samples from male HNC patients. The *IL32/ACE1* and *BIRC3/ACE1* mRNA ratios were correlated with the SMI (skeletal muscle index) of each male HNC patient. HNC patients were classified as sarcopenic when their SMI was below 52.4 cm^2^/m^2^. Comparison of the *IL32*/*ACE1* (A) and *BIRC3/ACE1* (B) mRNA ratios in muscle fragments from sarcopenic and non‐sarcopenic HNC male patients. Statistical analysis was performed using the Mann–Whitney *U* test (median values are indicated by horizontal bars). Statistical significance is indicated as follows: *p* < 0.05 (*). *p* < 0.05 was considered statistically significant. (C and D) Spearman correlation analysis between the SMI and *IL32*/*ACE1* or *BIRC3/ACE1* mRNA ratios in male HNC patients. *p* < 0.10 was considered statistically significant. The grey‐shaded area around the fitted regression line represents the 95% confidence interval (CI).

Regarding the abundances of *BIRC3* and *ACE1* mRNAs, as well as *IL32*/*ACE1* and *BIRC3*/*ACE1* mRNA ratios, we found no significant differences between sarcopenic and non‐sarcopenic patients (*BIRC3*: *p* = 0.9801, effect size *r* = 0.01; *ACE1*: *p* = 0.7927, effect size *r* = 0.05; IL32/ACE1: *p* = 0.1067, effect size *r* = 0.32; *BIRC3/ACE1*: *p* = 0.8672, effect size *r* = 0.04) (Figure S3). However, the values of SMI are usually spread over a greater range for men than for women. Therefore, to gain more sensitivity and to rule out any confusing effect of gender differences, we decided to focus our analysis on male HNC patients. In the male subgroup of HNC patients, the difference between sarcopenic and non‐sarcopenic subjects was significant for the *IL32/ACE1* but not the *BIRC3/ACE1* ratios (*IL32/ACE1*: *p* = 0.0133; effect size *r* = 0.94; *BIRC3/ACE1*: *p* = 0.9661; effect size *r* = 0.09) (Figure 3A,B). In addition, we found an inverse correlation between the values of the SMI and the *IL32*/*ACE1* but not the *BIRC3/ACE1* mRNA ratio (*IL32/ACE1*: *p* = 0.0926, *r* = −0.41, Spearman's correlation; *BIRC3/ACE1*: *p* = 0.6986, *r* = −0.10, Spearman's correlation) (Figure 3C,D).

## 5 Altered Transcription of *BIRC3*, *IL32* and *ACE1* Genes in Differentiating Myoblasts co‐Cultivated With Malignant HNC Cells

**FIGURE 4:**
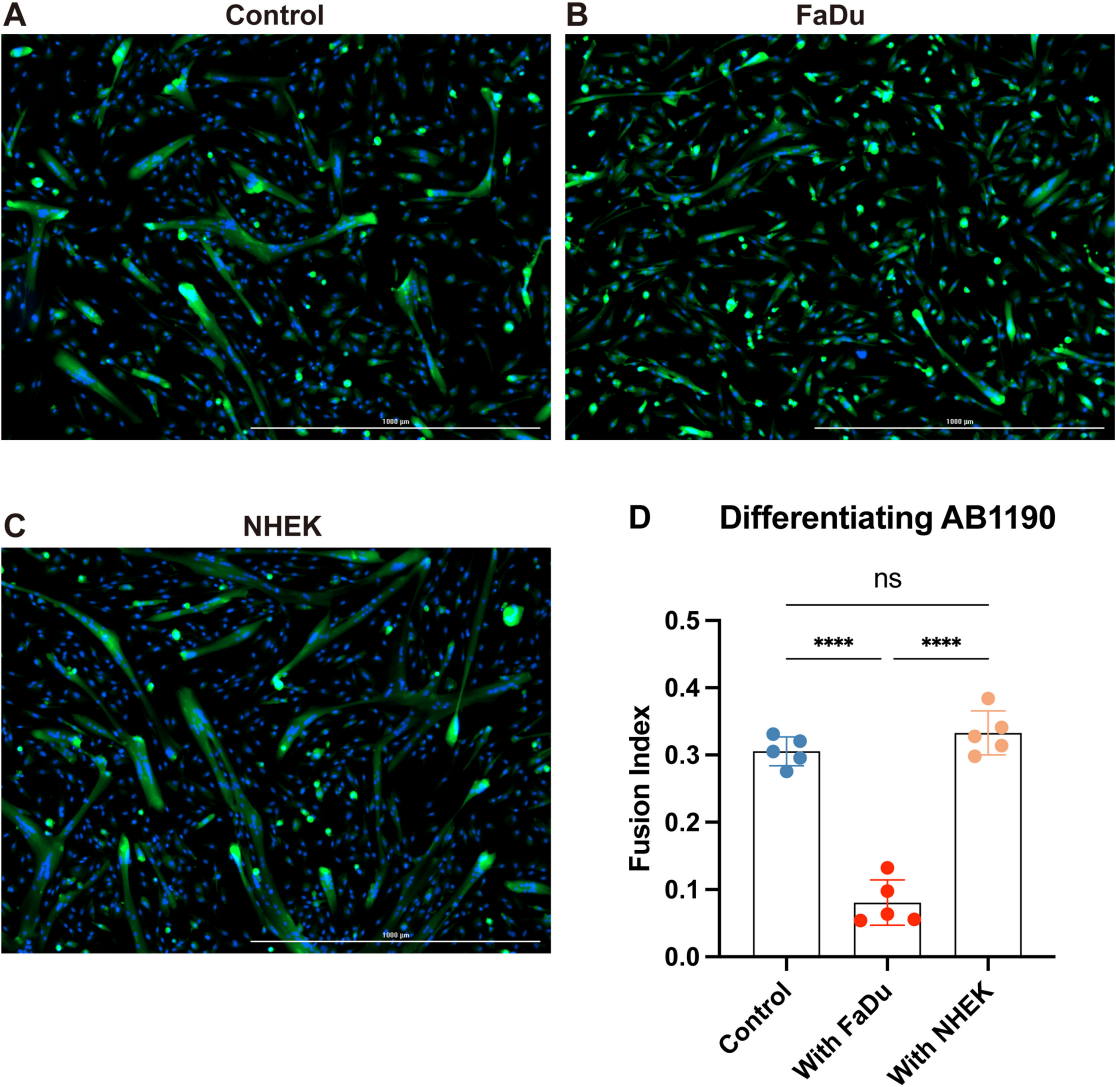
Assessment of myogenesis in AB1190 human myoblasts exposed to malignant or non‐malignant epithelial cells. Myogenic differentiation was induced in AB1190 myoblasts by switching to a differentiation medium (see Materials and Methods). Simultaneously, AB1190 cells were subjected to contactless co‐cultures with epithelial cells. AB1190 cells (target cells) were seeded in the lower compartments of Transwell devices (120,000 cells), while effector epithelial cells, either malignant (FaDu) or non‐malignant (NHEK), were seeded in the upper compartments (120,000 cells). After 4 days, AB1190 cells were stained with CellTracker to visualize the cytoplasm and Hoechst 33342 to label the nuclei. (A) Induction of myogenic differentiation in the absence of effector cells (control condition). Cell fusion and formation of myotubes are visible. (B) Induction of myogenic differentiation in the presence of FaDu cells. Few cell fusion events and myotubes are observed. (C) Induction of myogenic differentiation in the presence of NHEK cells. In contrast to the FaDu panel, there is no visible impairment of cell fusion and myotube formation. Scale bar: 1000 μm. (D) Graphical representation of the fusion indexes for the three conditions depicted in A, B and C. The fusion index was calculated as the percentage of nuclei within multinucleated myotubes (containing ≥ 2 nuclei) relative to the total number of nuclei. Statistical significance is indicated as follows: *p* < 0.0001 (****). *p* < 0.05 was considered statistically significant.

Our results obtained by transcription analysis of muscle fragments from HNC and control donors suggested that malignant HNC cells were able to exert a contactless influence on gene expression in muscle cells. To test this hypothesis, we performed indirect in vitro co‐culture experiments with HNC cells as effector cells and differentiating immortalised human myoblasts as target cells. We chose this experimental setting because in situ muscles are subjected to a permanent process of repair and regeneration with a switch from proliferation to differentiation being a critical step [27, 28]. AB1190 myoblasts were seeded in the lower compartment of Transwell plates [22]. The next day, FaDu carcinoma cells (or normal keratinocytes for control conditions) were seeded in the upper compartment of the experimental wells. Simultaneously, muscle cell differentiation was triggered by switching to differentiation medium as described in Materials and Methods. The same culture condition was maintained for 4 days prior to cell imaging, cell collection and RNA extraction. As shown in Figure 4, the differentiation of the AB1190 myoblasts was significantly inhibited by exposure to malignant epithelial cells (FaDu), whereas it was not affected by exposure to non‐malignant epithelial cells (NHEK).

**FIGURE 5:**
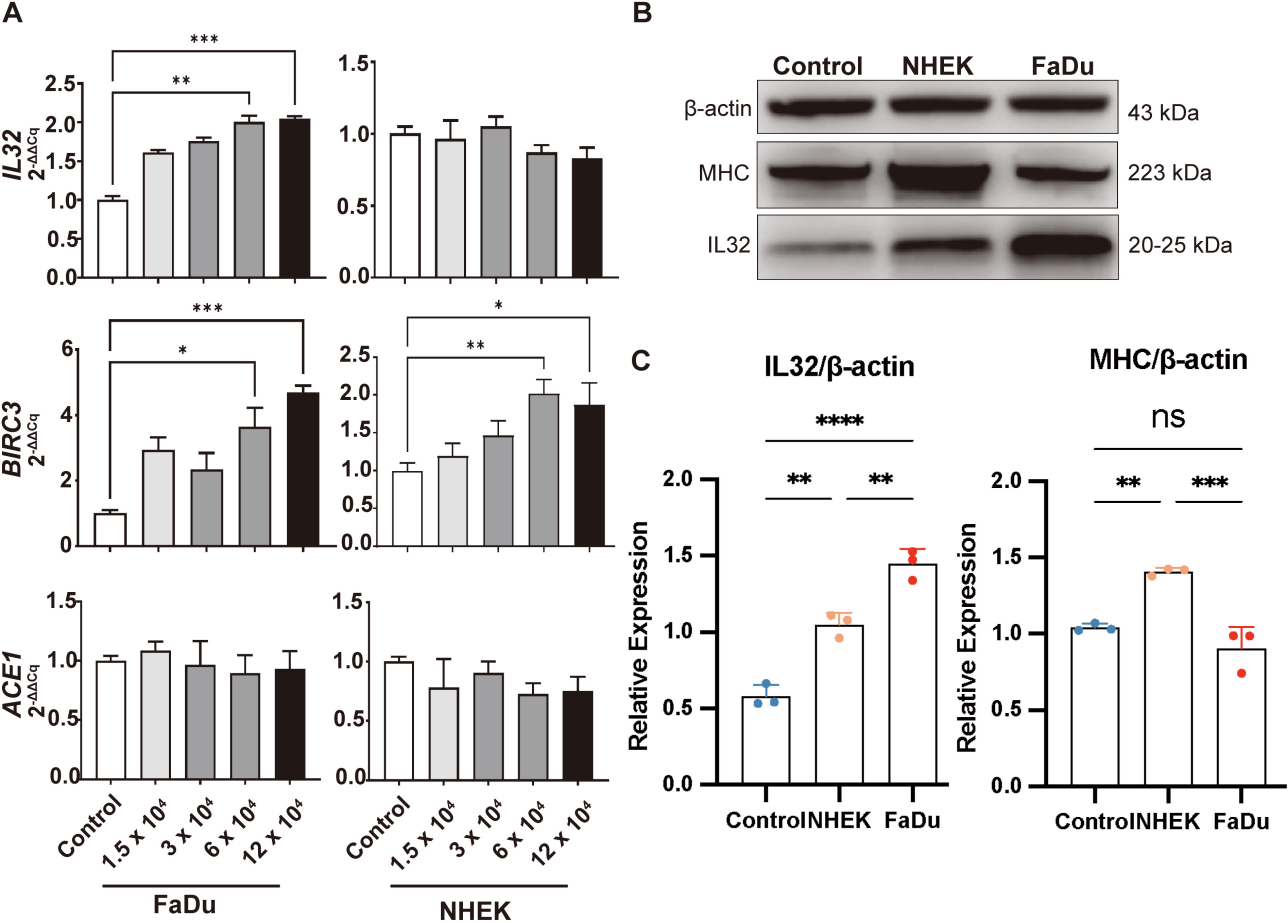
*IL32* and *BIRC3* mRNAs and IL32 protein in human muscle cells co‐cultivated with HNC cells. (A) AB1190 muscle cells were seeded into the lower compartment of a Transwell system at a constant density (120,000 cells/well). FaDu effector cells or NHEK (control) were seeded in the upper compartment at increasing densities (from zero cells/well for the control condition to 120,000 cells/well for maximal stimulation). Then myogenic differentiation was induced by adding the differentiation medium as described in Materials and Methods. Total RNA was extracted from muscle cells 4 days after the onset of myogenic differentiation. The relative concentrations of *IL32*, *BIRC3* and *ACE1* mRNAs were assessed by real time RT‐qPCR using the 2^−ΔΔCq^ method with the *PPIA* mRNA as the internal calibrator and the control condition as the external calibrator. Under exposure to FaDu cells, the concentrations of *IL32* and *BIRC3* mRNAs in muscle cells were consistently and substantially increased, without significant modifications of the amounts of *ACE1* transcripts. Under exposure to NHEK cells, there were trends towards greater concentrations of *BIRC3* mRNA and lower concentrations of *IL32* mRNA; again without significant changes for the *ACE1* mRNAs. The statistical analysis was done using the Kruskal–Wallis test. (B) Western blot detection of β‐actin, myosin heavy chain (MHC, a marker of myogenic differentiation) and IL32 in differentiating AB1190 myoblasts using three types of culture conditions like in (A): co‐culture with FaDu cells (malignant), co‐culture with NHEK cells (non‐malignant), absence of effector cells (control). (C) Quantification of IL32 and MHC expression levels normalised to β‐actin. The statistical analysis was done using the ANOVA test. Statistical significance is indicated as follows: *p* < 0.05 (*), *p* < 0.01 (**), *p* < 0.001 (***) and *p* < 0.0001 (****). *p* < 0.05 was considered statistically significant.

Next, target myoblasts were exposed to effector cells (FaDu or NHEK) at increasing cell densities from 1.5 × 10^4^ to 12 × 10^4^ per well. As shown in Figure 5A, the exposure of target myoblasts to FaDu cells resulted in a significant increase in the amounts of both *IL32* and *BIRC3* mRNAs. The amplitude of this increase was somehow proportional to the number of effector cells. In contrast, the exposure to NHEK cells did not increase *IL32* expression in AB1190 cells. There was even a small decrease for high amounts of effector cells. On the other hand, *BIRC3* expression was enhanced (again somehow reflecting the increase in the number of effector cells). Finally, neither FaDu nor NHEK significantly affected the amount of *ACE1* mRNA in AB1190 cells.

## 6 Up‐Regulation of the IL32 Protein in Differentiating Myoblasts Co‐Cultivated With Malignant HNC Cells

Because *IL32* mRNA was the most consistently and abundantly up‐regulated transcript in HNC muscle fragments, we sought to get more information on the expression of the corresponding protein using the co‐culture system and Western blot analysis (Figure 5B,C). IL32 exists in at least nine isoforms that arise from alternative splicing and differ in their inflammatory potency and cellular localisation [29]. In protein extracts from AB1190 myoblasts, the anti‐IL32 antibody detected a unique band in the range of 20–25 kDa; therefore, it is compatible with the α, β, γ or δ isoforms of IL32. This band was at its maximal intensity in AB1190 myoblasts stimulated with FaDu cells, followed by those stimulated with non‐malignant NHEK cells, while the control myoblasts exhibited the weakest IL32 signal. In contrast, the muscle differentiation marker MHC showed the lowest band intensity in FaDu‐stimulated myoblasts, an intermediate level in the control group and the highest intensity in NHEK‐stimulated myoblasts. This was consistent with the inhibition of myogenesis occurring in differentiating myoblasts exposed to the influence of FaDu cells (Figure 4).

## 7 Discussion

Cachexia and CRS are frequent events in HNC patients [10]. However, to our knowledge, no report has yet been published dealing with biological alterations of skeletal muscles occurring in this context. In our study, bulk RNAseq of muscle samples has revealed multiple transcriptional alterations in muscle fragments taken from HNC patients by comparison with healthy donors. At the next step, our investigations were focused on three mRNAs differentially expressed in the context of HNCs: *IL32* and *BIRC3* (up‐regulated) and *ACE1* (down‐regulated). Their differential expression was confirmed with statistical significance by RT‐qPCR on a relatively large set of muscle fragments: 29 HNC versus five control samples. *IL32* mRNA was the most abundant, about > 1000 higher than those of *BIRC3* and *ACE1* (Figures 3 and 4).

For practical and ethical reasons, we had to use muscular fragments from heterogeneous anatomic locations. For example, all control muscle samples were from the tensor fascia lata, whereas it was not the case for any HNC muscle sample. There was also heterogeneity inside our set of HNC muscle samples. Muscle fragments were from the sternocleidomastoid for most patients but from muscular flaps in a few cases (quadriceps femoris, pectoralis major etc.). Rarely, muscle fragments were available from both the sternocleidomastoid and muscular flaps. For a first evaluation of the impact of muscle anatomic location, we queried the database of a muscle transcriptome atlas [30]. The expressions of *IL32*, *BIRC3* and *ACE1* mRNAs were not found to be dependent on muscle types (https://tabbassidaloii.shinyapps.io/muscleAtlasShinyApp/). In addition, dendrograms and heatmaps of Figure 1C,D clearly demonstrate that the separation between HNC and control samples outweighs the variations related to the anatomical origin of the muscle fragments. Another technical issue was to ensure that *IL32* and *BIRC3* mRNAs detected in muscle fragments were mainly of muscular origin. We are confident that this was the case for two reasons: (i) tissue sections of these fragments showed overwhelming presence of muscular fibres (Figure S1); (ii) under exposure to malignant epithelial cells, *IL32* and *BIRC3* mRNA up‐regulation was observed in myoblasts grown in vitro that were at 100% of muscular origin (Figure 5A).

*BIRC3* encodes the c‐IAP2 protein. Inhibitors of apoptosis proteins (IAPs) are components of a highly conserved family of proteins involved in the regulation of apoptosis and necroptosis and in a large number of signalling pathways, often related to inflammation [31]. Three human IAPs—c‐IAP1, c‐IAP2 and XIAP—contain several baculovirus IAP repeat (BIR) and one ring functional domains. Both types of domains are involved in the control of cellular responses to TNF and TNF‐related cytokines. Both c‐IAP1 and c‐IAP2 are essential to prevent TNF‐induced cell death; each one is preferentially expressed in a specific set of tissues. According to the Human Protein Atlas, c‐IAP2 is not detected in healthy skeletal muscle, while the *BIRC3* mRNA is expressed at a very low level (https://www.proteinatlas.org/ENSG00000023445‐BIRC3/tissue). One previous in vitro study has shown that c‐IAP2 can protect arterial smooth muscle cells against apoptosis induced by collagen fragments [32]. It would be interesting to know whether this also applies to skeletal muscles. *ACE1* was selected for further investigations because of previous reports on its possible contribution to age‐related sarcopenia [25, 33]. However, in contrast with possible expectations, the concentrations of *ACE1* mRNA were lower in HNC than in control muscle samples, with the same low level for sarcopenic and non‐sarcopenic patients.

The main thrust of our study was on IL32 because its mRNA was more abundant than those of *BIRC3* and *ACE1*. In addition, according to a previous report, IL32 is suspected to be involved in obesity‐related sarcopenia [26]. IL32 was initially described as inducing TNF‐α and IL8 as well as TNF‐α and MIP‐2 expression, respectively, in human and murine monocytic cell lines [33]. The IL32 protein is abundant in immune cells and to a lesser extent in various organs like the lungs, colon, heart and brain [33]. IL32 exists in at least nine isoforms that arise from alternative splicing and differ in their inflammatory potency and cellular localisation. Some isoforms of IL32 are secreted, and some are intracellular modulators of transduction and inflammation. IL32 can interact with cytoplasmic proteins like PKC delta or paxillin and FAK1 [33, 34]. IL32 can enhance the immune response, for example, by inducing the production of interferon‐γ by NK cells [35]. Nevertheless, it can also be immunosuppressive for example, by promoting macrophage M2 polarisation [36, 37]. IL32 is highly expressed in various tumours, but with distinct effects depending on the tumour type. It promotes the progression of liver, pancreatic and lung cancer, whereas it has anti‐tumour functions in colon, prostate and cervical cancer [38].

As previously mentioned, Davegårdh et al. have reported the high expression of IL32 in skeletal muscles in the context of obesity‐related sarcopenia. Consistently, transgenic mice ectopically expressing human IL32 in skeletal muscles had a reduced tibialis anterior muscle weight compared to the control animals as well as a higher plasma glucose level and poor insulin response [26]. The same study reported a possible link between the expression of the IL32 protein and myoblast differentiation. In contrast, our in vitro studies do not support the hypothesis of a link between the induction of IL32 and the process of muscular differentiation, at least in the context of HNCs. Indeed, under exposure of differentiating myoblasts to FaDu cells, the enhanced expression of IL32 mRNA and protein occurred despite the impairment of myogenesis. Reciprocally, under exposure to NHEK cells, IL32 expression remains low at the mRNA level and minimally increases at the protein level despite morphological and biochemical evidence of complete muscular differentiation. Consistently, the data obtained from clinical muscle samples do not support a connection between the induction of *IL32* mRNA and myogenesis. It is known that mature muscle fibres constitute 95%–97% of muscle [39]. Therefore, the differences in the abundance of *IL32* mRNA detected in sarcopenic and non‐sarcopenic muscles are not likely to reflect differences in the overall level of differentiation.

Our data have shown some influence of patient gender on *IL32* expression in muscle fragments (Figure S4). However, multivariate analysis showed that sarcopenia remained a more robust predictor of *IL32* mRNA expression than patient gender or age as well as tumour volume (see Supplementary File S1). When analysis was restricted to male patients, the *IL32*/*ACE1* ratio was significantly elevated in sarcopenic individuals adding further evidence of a correlation with sarcopenia independent of patient gender (Figure 3).

In summary, we provide evidence that HNC cells can remotely modulate skeletal muscle gene expression, potentially contributing to CRS. In particular, *IL32* transcripts were much more abundant in muscle fragments from HNC patients than healthy donors. Therefore, the up‐regulation of *IL32* mRNA emerges as a potential signature of the remote influence of malignant cells on skeletal muscles. This hypothesis is supported by in vitro experiments. Exposure of differentiating myoblasts to malignant HNC cells (FaDu) induced up‐regulation of *IL32* and *BIRC3* mRNAs, whereas *IL32* mRNA was not up‐regulated in myoblasts exposed to non‐malignant epithelial cells (NHEK). The dose‐dependent transcriptional response to FaDu cells supports the hypothesis of a soluble factor involved in muscle gene deregulation. Finally, as previously mentioned, a substantial increase of IL32 protein expression parallel to the up‐regulation of IL32 mRNA was detected in myoblasts exposed to FaDu cells.

These results open several perspectives. First, detection of IL32 mRNA and protein in muscle fragments might pave the way for their use as tissue biomarkers of muscle deterioration and dysfunction in the context of HNCs. This will require a more systematic characterisation of muscular IL32 isoforms expressed in this pathological context. It will also be useful to include parameters of muscle alterations complementary to volumetric indices, for example, the handgrip test. Our attempt to detect IL32α in plasma samples from HNC patients has been disappointing (Supplementary File S2 and Supplementary Figure S5). Development of novel ELISA tests, especially tests reacting with various IL32 isoforms, will be necessary before drawing definitive conclusions. Another perspective will be to investigate the contribution of IL32 in the biological disorders underlying the dysfunctions of muscle cells, for example, inflammation, impairment of myogenesis or metabolic disorders.

The methodological repercussions of our study will be as important or even more important than our insights on molecular alterations of skeletal muscles in the context of HNCs. It is very encouraging to see that at least one of these alterations, namely, the increased amount of *IL32* mRNA was replicated in vitro using immortalised myoblasts co‐cultivated with malignant cells. Indeed, this experimental system could easily be enriched by multiple additions and improvements. For example, on the side of effector cells, it will be possible to use tumour cells in 2D or 3D primary cultures and to combine the malignant cells with tumour‐infiltrating cells like macrophages or cancer‐associated fibroblasts. The same will apply to the muscle side of the system. It will be possible to use muscle cells in primary culture and/or to mix them with various types of leukocytes as well as with adipocytes or fibroblasts.

This experimental system will also be quite useful for the identification of the molecular effectors supporting the distant effects of malignant cells on skeletal muscles, for example, soluble proteins or microRNAs. For this aim, various types of conditioned media collected on both sides of the co‐cultivation device will be subjected to analytical procedures, for example, mass spectrometry or RNAseq. At the next step, this system might also become useful to test biomolecules designed to neutralize tumour‐driven mechanisms of sarcopenia (e.g., antibodies or aptamers). Overall, using these approaches, we expect to significantly reduce the need for animal experimentation aiming at the biological elucidation of CRS and the design of appropriate therapeutic tools.

## Funding

The experimental work was supported by a grant from ‘Entreprises contre le Cancer’ (GEFLUC—ECC2021). The Magnolia trial (NCT4842162 and EudraCT 2019‐002442‐20) was supported by a PHRC grant from the Institut National du Cancer (INCa) (2016). Fei Chen is the recipient of a fellowship from the Chinese Scholarship Council. Yegor Vassetzky was supported by the government program of basic research in Koltzov Institute of Developmental Biology No. 0088‐2024‐0010. Overall, this study was supported by the Centre National de la Recherche Scientifique, Université Paris‐Saclay and Institut Gustave Roussy in terms of staff, infrastructure and supply.

## Ethics Statement

All human studies have been approved by the French Ethics committee ‘Sud‐Est V’ on February 13, 2020, and have therefore been performed in accordance with the ethical standards laid down in the 1964 Declaration of Helsinki and its later amendments.

## Conflicts of Interest

Imane Baïche, Héla Hachicha, Thierry Ragot, Céline Gracia, Aurore Gelin, Anaïs Gader, Caroline Even, Ingrid Breuskin, Odile Casiraghi, Filippo Dall'Ollio, Catherine Brenner, Karim Benihoud, Thibault Dayris, Yegor Vassetzky, François Bidault, Philippe Gorphe, Pierre Busson and Fei Chen declare that they have no conflicts of interest.

## Supporting information


**Figure S1:** Haematoxylin and eosin (H&E) staining of skeletal muscle tissues from three HNC patients. Representative sections from sternocleidomastoid fragments are shown for patients HNC25 (left), HNC26 (middle) and HNC27 (right). Muscle fibre morphology, size variation and overall tissue architecture can be appreciated. Scale bar: 100 μm.

**Figure S2:** Correlation between values of SMI based on either direct measurement of paravertebral muscle surface at L3 level or measurement at C3 followed by extrapolation to L3. Fifteen HNC patients with CT images available at both the C3 and L3 vertebral levels were included in this comparison. Paravertebral muscle surface (called cross‐sectional area [CSA]) was assessed at the C3 and L3 levels using the Horos software (for more details, see Materials and Methods). The CSA from C3 was processed using the Swartz algorithm (Reference 18) to give an extrapolated L3 CSA. Finally, for each patient, two SMI values were calculated using either the direct or the extrapolated value of the L3 CSA. The correlation between the two SMI values is demonstrated using Pearson analysis. *p* < 0.05 was considered statistically significant.

**Figure S3:** jcsm70160‐sup‐0003‐Supplementary_FigureS3.tiff. *IL32*, *BIRC3* and *ACE1* mRNAs in muscle samples from sarcopenic and non‐sarcopenic HNC patients. The amounts of *IL32*, *BIRC3* and *ACE1* mRNAs as well as the *IL32/ACE1* and *BIRC3/ACE1* mRNA ratios were analysed in connection with the SMI (skeletal muscle index) of HNC patients. Male and female HNC patients were classified as sarcopenic when their SMI was below 52.4 and 38.5 cm^2^/m^2^, respectively. Comparison of the amounts of *IL32* (A), *BIRC3* (B) and *ACE1* (C) mRNAs, in muscle samples from sarcopenic and non‐sarcopenic HNC patients (male and female) using PPIA mRNA as the internal calibrator. Comparison of *IL32*/*ACE1* (D) and *BIRC3/ACE1* (E) mRNA ratios in muscle fragments from sarcopenic and non‐sarcopenic HNC patients (male and female) (for these comparisons, the *ACE1* mRNA is the internal calibrator). Statistical analysis was performed using the Mann–Whitney *U* test (median values are indicated by horizontal bars). Statistical significance is indicated as follows: *p* < 0.05 (*). *p* < 0.05 was considered statistically significant.

**Figure S4:** jcsm70160‐sup‐0004‐Supplementary_FigureS4.tiff. *IL32*, *BIRC3* and *ACE1* mRNAs in muscle samples from male and female HNC patients. Comparison of the amounts of *IL32* (A), *BIRC3* (B) and *ACE1* (C) mRNAs, in muscle samples from male and female HNC patients (using *PPIA* mRNA as the internal calibrator). Statistical analysis was performed using the Mann–Whitney *U* test (median values are indicated by horizontal bars). Statistical significance is indicated as follows: *p* < 0.05 (*). *p* < 0.05 was considered statistically significant.

**Figure S5:** IL32 in plasma samples from HNC patients and control donors. (A) IL32 plasma concentrations in HNC patients and control donors. Its detection was made using a commercial ELISA reacting with the IL32α isoform. (B) IL32 plasma concentrations in sarcopenic and non‐sarcopenic HNC patients. Statistical significance is indicated as follows: *p* < 0.05 (*). Statistical analysis was performed using the Mann–Whitney *U* test (median values are indicated by horizontal bars). (C) Spearman correlation analysis between SMI and plasma IL32 concentrations in HNC patients. The grey‐shaded area around the fitted regression line represents the 95% confidence interval (CI).

**Table S1:** Inclusion and exclusion criteria for the Magnolia protocol.

**Table S2:** Clinical contexts and anatomic origins of muscle fragments used for RNAseq analysis (HNC‐RNAseq series and corresponding controls).

**Table S3:** Primer pairs used for PCR analysis.

**Table S4:** Small tandem repeat analysis of cell lines used in this study.

**Table S5:** Count table of comparative bulk RNAseq for HNC and control muscles.

**Data S1:** Supplementary Information.

**Data S2:** Supplementary Information.

**Data S3:** Supplementary Information.

**Data S4:** Supplementary Information.
