# Correction to ‘Enhanced Expression of *IL32* mRNA in Skeletal Muscles in the Context of Head and Neck Carcinomas’

**DOI:** 10.1002/jcsm.70239

**Published:** 2026-02-27

I. Baïche , H. Hachicha , T. Ragot , et al., “Enhanced Expression of *IL32* mRNA in Skeletal Muscles in the Context of Head and Neck Carcinomas” Journal of Cachexia, Sarcopenia and Muscle 17, no. 1 (2026): e70160, 10.1002/jcsm.70160.41457346 PMC12745337

In the list of authors, the name ‘Filippo Dall'Ollio’ was misspelled. It should be corrected to ‘**Filippo Dall'Olio**’.

We apologize for this error.

