# Supplementary figures and images for: Enhanced Expression of IL32 mRNA in Skeletal Muscles in the Context of Head and Neck Carcinomas
Source: J Cachexia Sarcopenia Muscle. 2025 Dec 28;17(1):e70160. doi: 10.1002/jcsm.70160 (PMC12745337; doi:10.1002/jcsm.70160)

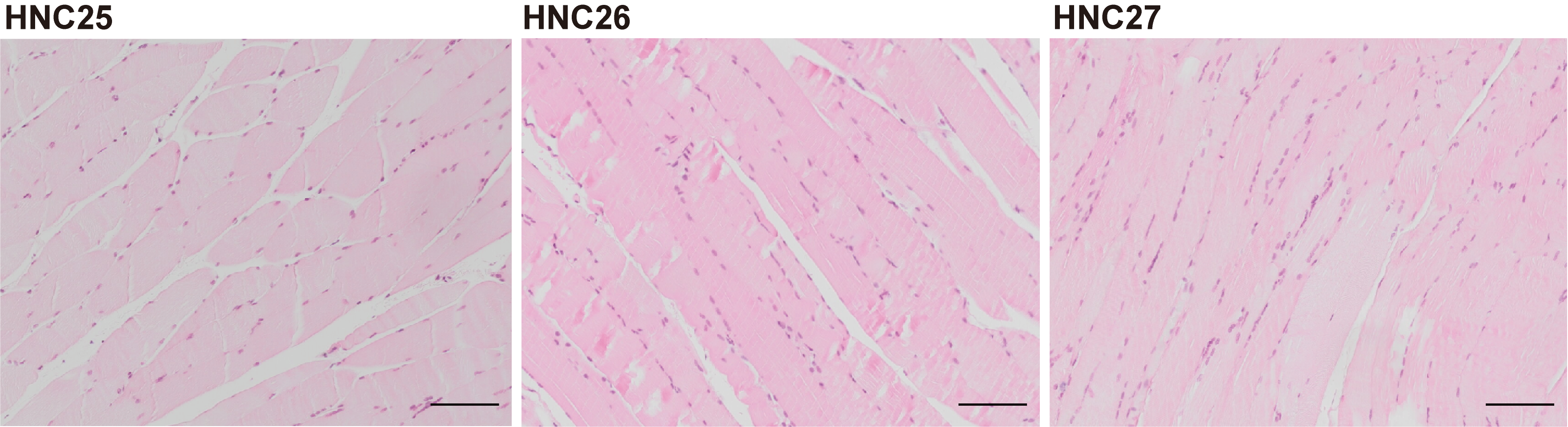

Supplement: Supplementary file 1 — Figure S1: Haematoxylin and eosin (H&E) staining of skeletal muscle tissues from three HNC patients. Representative sections from sternocleidomastoid fragments are shown for patients HNC25 (left), HNC26 (middle) and HNC27 (right). Muscle fibre morphology, size variation and overall tissue architecture can be appreciated. Scale bar: 100 μm. [file JCSM-17-e70160-s009.tif]

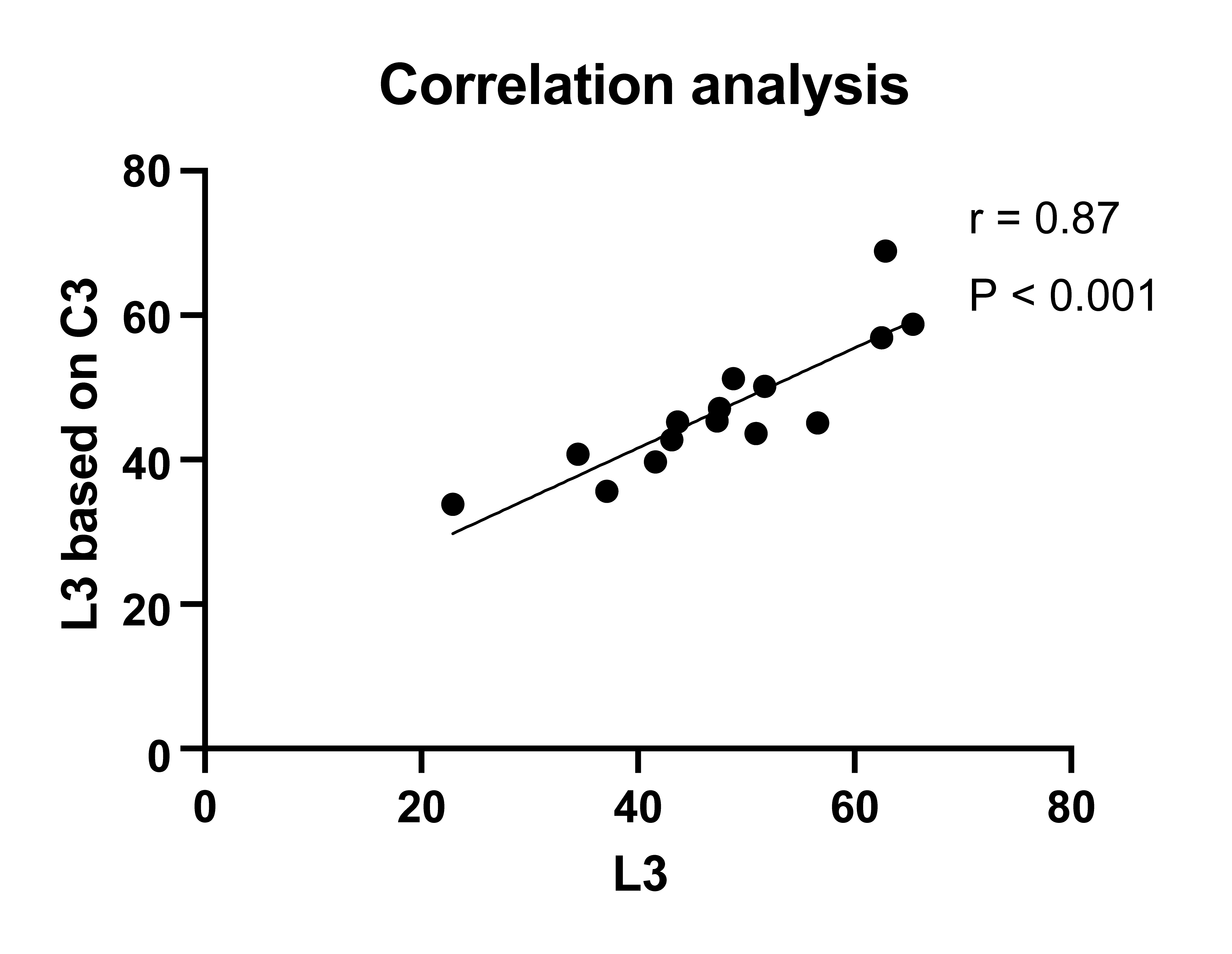

Supplement: Supplementary file 2 — Figure S2: Correlation between values of SMI based on either direct measurement of paravertebral muscle surface at L3 level or measurement at C3 followed by extrapolation to L3. Fifteen HNC patients with CT images available at both the C3 and L3 vertebral levels were included in this comparison. Paravertebral muscle surface (called cross‐sectional area [CSA]) was assessed at the C3 and L3 levels using the Horos software (for more details, see Materials and Methods). The CSA from C3 was processed using the Swartz algorithm (Reference 18) to give an extrapolated L3 CSA. Finally, for each patient, two SMI values were calculated using either the direct or the extrapolated value of the L3 CSA. The correlation between the two SMI values is demonstrated using Pearson analysis. p < 0.05 was considered statistically significant. [file JCSM-17-e70160-s005.tif]

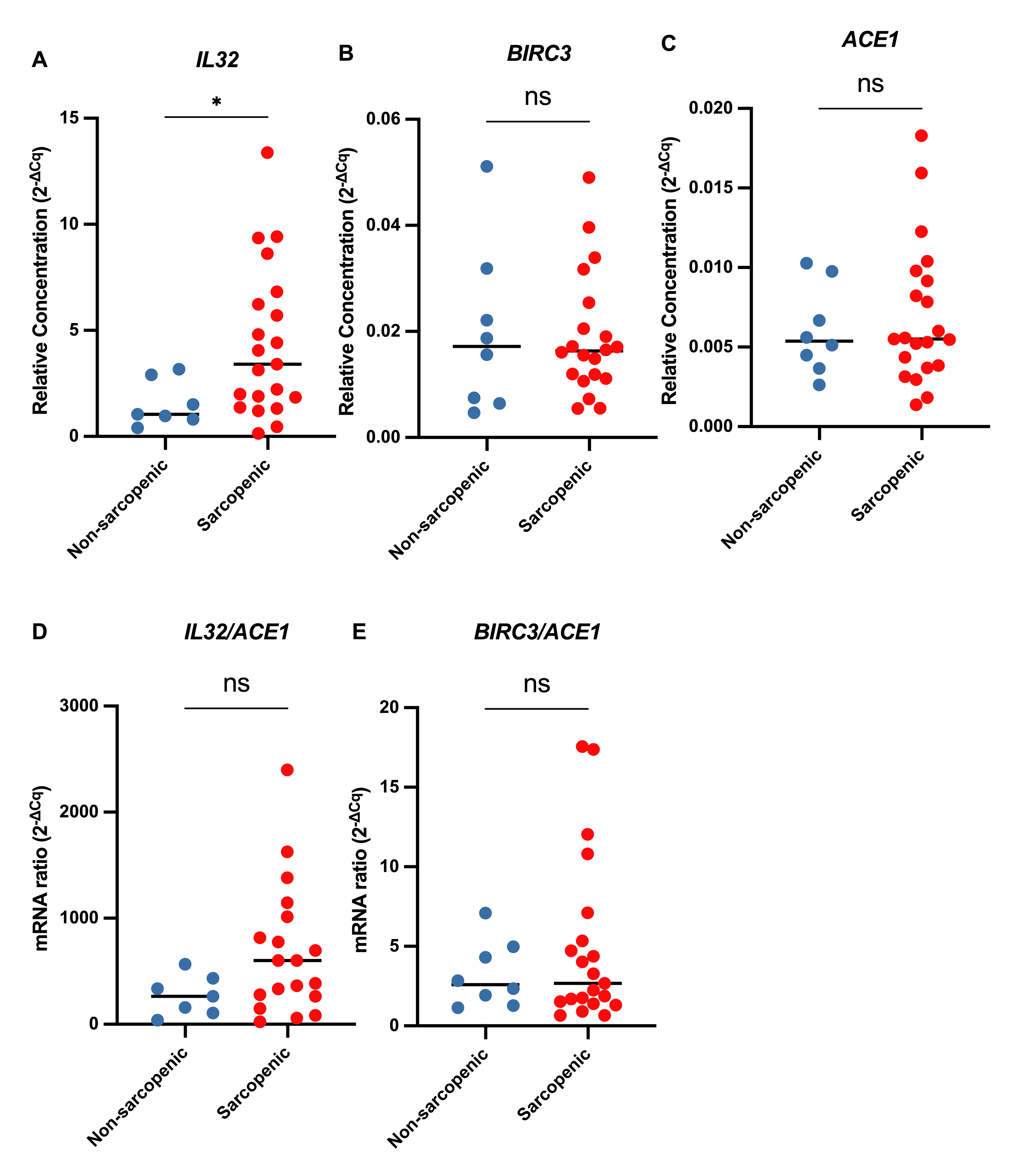

Supplement: Supplementary file 3 — Figure S3: jcsm70160‐sup‐0003‐Supplementary_FigureS3.tiff. IL32, BIRC3 and ACE1 mRNAs in muscle samples from sarcopenic and non‐sarcopenic HNC patients. The amounts of IL32, BIRC3 and ACE1 mRNAs as well as the IL32/ACE1 and BIRC3/ACE1 mRNA ratios were analysed in connection with the SMI (skeletal muscle index) of HNC patients. Male and female HNC patients were classified as sarcopenic when their SMI was below 52.4 and 38.5 cm2/m2, respectively. Comparison of the amounts of IL32 (A), BIRC3 (B) and ACE1 (C) mRNAs, in muscle samples from sarcopenic and non‐sarcopenic HNC patients (male and female) using PPIA mRNA as the internal calibrator. Comparison of IL32/ACE1 (D) and BIRC3/ACE1 (E) mRNA ratios in muscle fragments from sarcopenic and non‐sarcopenic HNC patients (male and female) (for these comparisons, the ACE1 mRNA is the internal calibrator). Statistical analysis was performed using the Mann–Whitney U test (median values are indicated by horizontal bars). Statistical significance is indicated as follows: p < 0.05 (*). p < 0.05 was considered statistically significant. [file JCSM-17-e70160-s003.tiff]

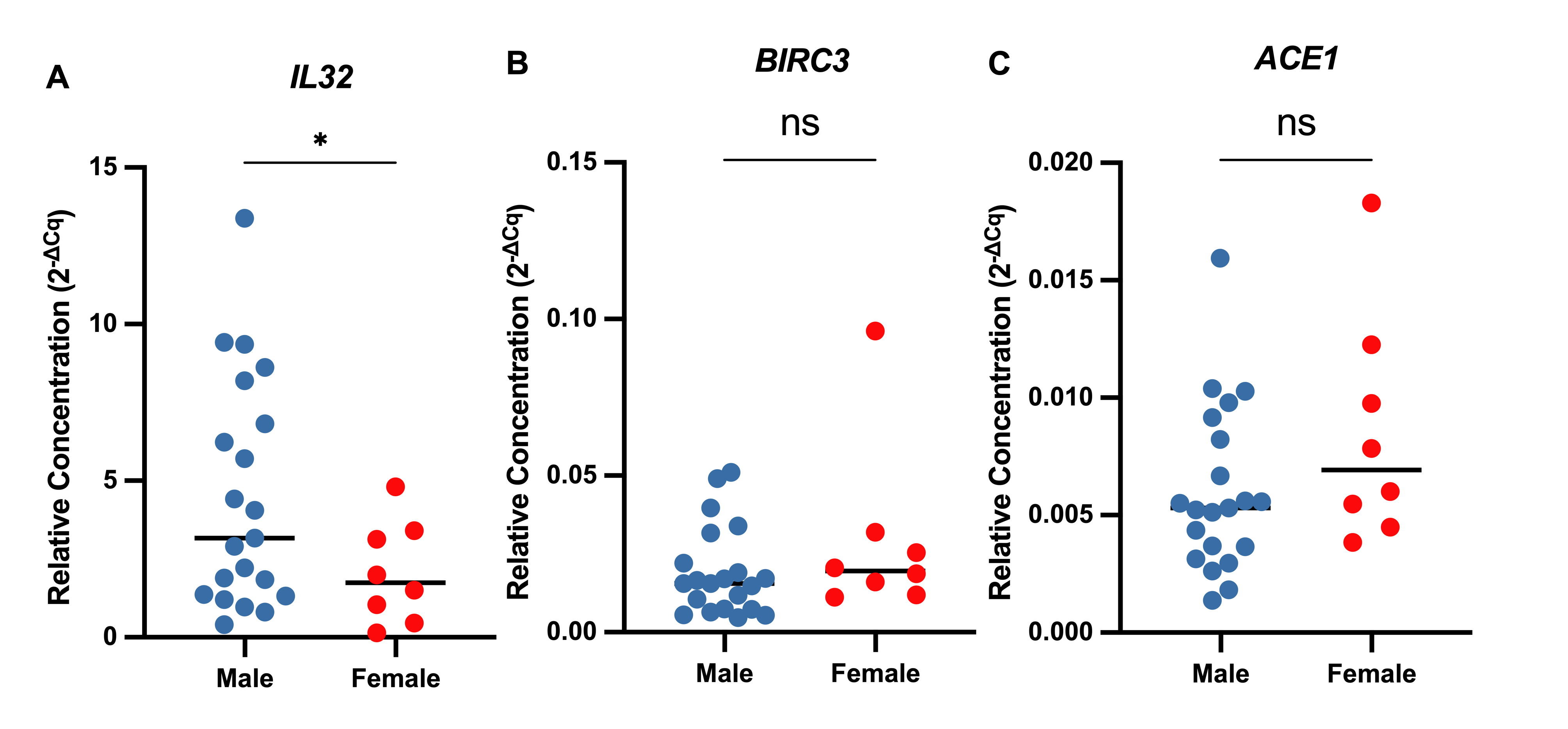

Supplement: Supplementary file 4 — Figure S4: jcsm70160‐sup‐0004‐Supplementary_FigureS4.tiff. IL32, BIRC3 and ACE1 mRNAs in muscle samples from male and female HNC patients. Comparison of the amounts of IL32 (A), BIRC3 (B) and ACE1 (C) mRNAs, in muscle samples from male and female HNC patients (using PPIA mRNA as the internal calibrator). Statistical analysis was performed using the Mann–Whitney U test (median values are indicated by horizontal bars). Statistical significance is indicated as follows: p < 0.05 (*). p < 0.05 was considered statistically significant. [file JCSM-17-e70160-s007.tiff]

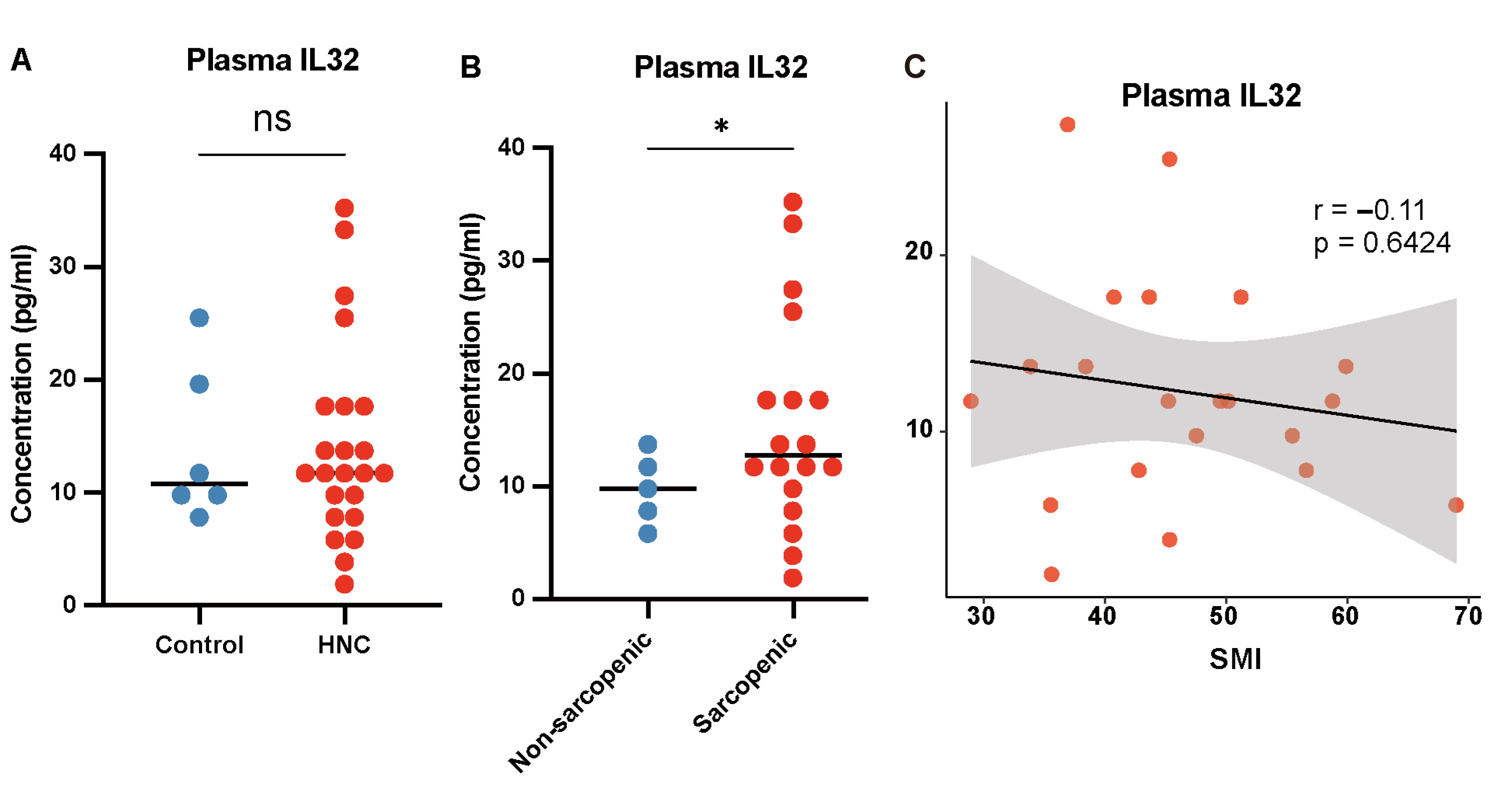

Supplement: Supplementary file 5 — Figure S5: IL32 in plasma samples from HNC patients and control donors. (A) IL32 plasma concentrations in HNC patients and control donors. Its detection was made using a commercial ELISA reacting with the IL32α isoform. (B) IL32 plasma concentrations in sarcopenic and non‐sarcopenic HNC patients. Statistical significance is indicated as follows: p < 0.05 (*). Statistical analysis was performed using the Mann–Whitney U test (median values are indicated by horizontal bars). (C) Spearman correlation analysis between SMI and plasma IL32 concentrations in HNC patients. The grey‐shaded area around the fitted regression line represents the 95% confidence interval (CI). [file JCSM-17-e70160-s004.tif]
