# Supplementary material for: Enhanced Expression of IL32 mRNA in Skeletal Muscles in the Context of Head and Neck Carcinomas
Source: J Cachexia Sarcopenia Muscle. 2025 Dec 28;17(1):e70160. doi: 10.1002/jcsm.70160 (PMC12745337; doi:10.1002/jcsm.70160)
Supplement: Supplementary file 6 — Table S1: Inclusion and exclusion criteria for the Magnolia protocol. [file JCSM-17-e70160-s006.docx]

**Supplementary Table 1. Inclusion and exclusion criteria for the Magnolia protocol**

| **Inclusion Criteria** | **Exclusion Criteria** |
| --- | --- |
| - Male or female aged 18 years or older | - Severe medical comorbidities or contraindications to radiotherapy or surgery |
| - ECOG 0-2 | - Inoperable primary tumor |
| - Histological confirmation of squamous cell carcinoma | - History of head and neck cancer within the last 5 year |
| - Location of the primary tumor in the oral cavity or oropharynx | - History of radiotherapy for head and neck cancer |
| - Disease operable with the intention of complete resection | - Metastatic cancer |
| - The patient must be affiliated with a social security plan or a beneficiary of a similar plan | - Tumors showing areas of necrosis on preoperative imaging |
| - The patient must understand, sign, and date the written consent form prior to any protocol-specific procedure. The patient must be able and willing to comply with study visits and procedures described in the protocol. | - History of invasive cancer unless there has been no recurrence for more than 5 years, with the exception of non-melanoma skin cancers - Pregnant or breastfeeding women |
