# Supplementary material for: Enhanced Expression of IL32 mRNA in Skeletal Muscles in the Context of Head and Neck Carcinomas
Source: J Cachexia Sarcopenia Muscle. 2025 Dec 28;17(1):e70160. doi: 10.1002/jcsm.70160 (PMC12745337; doi:10.1002/jcsm.70160)
Supplement: Supplementary file 7 — Table S2: Clinical contexts and anatomic origins of muscle fragments used for RNAseq analysis (HNC‐RNAseq series and corresponding controls). [file JCSM-17-e70160-s014.docx]

**Supplementary Table 2. Clinical contexts and anatomic origins of muscle fragments used for RNAseq analysis (HNC-RNAseq series and corresponding controls)**

| **Subject** | **Sex** | **Age** | **Muscle Type** | **SMI*** | **Cancer-related Sarcopenia**** |
| --- | --- | --- | --- | --- | --- |
| Ctrl A | F | 60 | *Tensor Fascia Lata* | - | - |
| Ctrl B | M | 63 | *Tensor Fascia Lata* | - | - |
| Ctrl C | F | 59 | *Tensor Fascia Lata* | - | - |
| HNC1 | F | 88 | Sternocleidomastoid | 29.0 | yes |
| HNC2 | M | 53 | *Pectoralis major* | 45.3 | yes |
|  |  |  | Sternocleidomastoid |  |  |
| HNC3 | M | 66 | Sternocleidomastoid | 35.6 | yes |
|  |  |  | *Pectoralis major* |  |  |
| HNC4 | M | 76 | Sternocleidomastoid | 43.5 | yes |
|  |  |  | *Quadriceps femoris* |  |  |
| HNC5 | F | 72 | *Latissimus dorsi* | 39.7 | no |
| HNC6 | M | 63 | Digastric | 49.5 | yes |

* at L3 (based on C3 CT scan)

** as explained in the Materials and Methods section, the threshold for cancer-related sarcopenia was set at 52.4 cm²/m² and 38.5 cm²/m² for male and female patients, respectively.
