# Supplementary material for: Enhanced Expression of IL32 mRNA in Skeletal Muscles in the Context of Head and Neck Carcinomas
Source: J Cachexia Sarcopenia Muscle. 2025 Dec 28;17(1):e70160. doi: 10.1002/jcsm.70160 (PMC12745337; doi:10.1002/jcsm.70160)
Supplement: Supplementary file 8 — Table S3: Primer pairs used for PCR analysis. [file JCSM-17-e70160-s010.docx]

**Supplementary Table 3. Primer pairs used for PCR analysis.**

| **Gene** | **Forward** | **Reverse** |
| --- | --- | --- |
| *IL-32** | 5′-GTCATCTCAGGCCTTGGCTC-3′ | 5′-TTCGGGCCTTCAGCTTCTTC-3′ |
| *BIRC3* | 5’-GCTTTTGCTGTGATGGTGGACTC-3’ | 5’-CTTGACGGATGAACTCCTGTCC-3’ |
| *ACE1* | 5’-CCGGCAACTTTTCTGCTGACG-3’ | 5’-GATGTTGGTGTCGTGCGCC-3’ |
| *PPIA* | 5’-GGCAAATGCTGGACCCAACACA-3’ | 5’-TGCTGGTCTTGCCATTCCTGGA-3’ |

*Primers matching with β, γ and δ isoforms
