# Supplementary material for: Enhanced Expression of IL32 mRNA in Skeletal Muscles in the Context of Head and Neck Carcinomas
Source: J Cachexia Sarcopenia Muscle. 2025 Dec 28;17(1):e70160. doi: 10.1002/jcsm.70160 (PMC12745337; doi:10.1002/jcsm.70160)
Supplement: Supplementary file 9 — Table S4: Small tandem repeat analysis of cell lines used in this study. [file JCSM-17-e70160-s011.docx]

**Supplementary Table 4. Small Tandem Repeat analysis of cell lines used in this study**

| Cell line | FaDu* | AB1190** |
| --- | --- | --- |
| D8S1179 | 13, 13 | 13, 15 |
| D21S11 | 31.2, 31.2 | 30, 31 |
| D7S820 | 11, 12 | 10, 11 |
| CSF1PO | 12, 12 | 9, 12 |
| D3S1358 | 17, 18 | 17, 18 |
| TH01 | 8, 8 | 7, 9.3 |
| D13S317 | 8, 9 | 11, 14 |
| D16S539 | 11, 11 | 12, 13 |
| D2S1338 | 19, 19 | 19, 21 |
| D19S433 | 14, 16 | 14, 15.2 |
| vWA | 15, 17 | 16, 17 |
| TPOX | 11, 11 | 8, 12 |
| D18S51 | 16, 16 | 16, 17 |
| AMEL | * | X, Y |
| D5S818 | 12, 12 | 12, 13 |
| FGA | 25, 25 | 22, 26 |

* STR profile identical with Cellosaurus CVCL_1218 (wild-type FaDu)

** This STR profile is characteristic of a homogeneous human cell line distinct from all cell lines listed in databases
