# Supplementary material for: Enhanced Expression of IL32 mRNA in Skeletal Muscles in the Context of Head and Neck Carcinomas
Source: J Cachexia Sarcopenia Muscle. 2025 Dec 28;17(1):e70160. doi: 10.1002/jcsm.70160 (PMC12745337; doi:10.1002/jcsm.70160)
Supplement: Supplementary file 11 — Data S1: Supplementary Information. [file JCSM-17-e70160-s008.docx]

**Supplementary File 1**

**Multivariate analysis of factors with potential effects on *IL32* mRNA expression in muscle samples**

Age was not significantly associated with *IL32* mRNA expression (β = −0.018, 95% CI: −0.051 to 0.018, P = 0.333), male gender and tumor volume showed a positive but non-significant association (β = 0.572, 95% CI: −0.303 to 1.447, P = 0.213; β = 0.870, 95% CI: −0.039 to 1.700, P = 0.840). In contrast, sarcopenia/non sarcopenia was significantly associated with increased *IL32* mRNA levels (β = 0.816, 95% CI: 0.038 to 1.670, P = 0.073 – threshold 10%).
