# Supplementary material for: Enhanced Expression of IL32 mRNA in Skeletal Muscles in the Context of Head and Neck Carcinomas
Source: J Cachexia Sarcopenia Muscle. 2025 Dec 28;17(1):e70160. doi: 10.1002/jcsm.70160 (PMC12745337; doi:10.1002/jcsm.70160)
Supplement: Supplementary file 12 — Data S2: Supplementary Information. [file JCSM-17-e70160-s013.docx]

**Supplementary File 2**

**Detection of IL32α in plasma samples from HNC patients**

**Materials and Methods**

Blood samples were collected intra-operatively on EDTA in the frame of the Magnolia protocol (NCT04842162). Plasma was separated from blood cells by centrifugation at 1700 g, for 15 min at 4°C and stored at minus 80°C, with a delay not exceeding 2h following blood collection. IL32α was measured in the plasma samples by Eve Technologies (Calgary, Canada) using the following kit: MILLIPLEX® Human Cytokine/Chemokine Magnetic Bead Panel IV, Cat. #HCYP4MAG-64K (Millipore-Sigma, Burlington, Massachusetts, USA).

**Results**

We were wondering whether the up regulation of IL32 in skeletal muscles had some repercussions on IL32 in plasma samples from HNC patients. As a first step to address this question, we had recourse to a commercial ELISA that was available for IL32α, one isoform potentially detected in human myoblasts by western blot (Figure 5). We used this assay to investigate plasma samples that were available for 26 out of 29 patients of the PCR-HNC-series (Table 2; HNC plasma series). Plasma samples from six healthy donors were used as controls (Table 2). The concentration of IL32α was very low in all samples. The median plasma concentrations were similar for HNC patients and control donors (P = 0.9672, effect size r = 0.01) (Figure S5A). However, among HNC patients (including both males and females), we found a greater median concentration for sarcopenic vs. non-sarcopenic subjects (P = 0.0438, effect size r = 0.64) (Figure S5B). In addition, there was a weak inverse correlation between the SMI and the concentration of plasma IL32 (P = 0.6424, r = -0.11, Spearman’s correlation) (Figure S5C).

**Discussion**

Willing to investigate IL32 in plasma samples of HNC patients, we used an ELISA reacting with the α isoform. We found no significant differences in its plasma concentration between HNC patients and control donors. This is suggesting that IL32α is not secreted into blood by muscles. It is consistent with previous knowledge suggesting that IL32α is less easily secreted than other IL32 isoforms [34]. However, sarcopenic patients had higher IL32 plasma levels than non-sarcopenic patients, though without robust correlation to muscle mass indices. This could be explained by muscle degradation and damage in sarcopenic patients.
