# Supplementary material for: Enhanced Expression of IL32 mRNA in Skeletal Muscles in the Context of Head and Neck Carcinomas
Source: J Cachexia Sarcopenia Muscle. 2025 Dec 28;17(1):e70160. doi: 10.1002/jcsm.70160 (PMC12745337; doi:10.1002/jcsm.70160)
Supplement: Supplementary file 13 — Data S3: Supplementary Information. [file JCSM-17-e70160-s012.docx]

**Supplementary Materials and Methods**

**Bulk RNA sequencing and bioinformatics analysis**

RNA samples were processed at the Next Generation Sequencing facility of the Curie Institute (ICGEX - Paris). Sequencing was performed using a NovaSeq 6000 instrument (Illumina) to generate 100 base-pair paired-end reads, with approximately 60 million reads per sample.

Raw read quality was assessed using FastQC (default parameters). Adapter trimming and quality filtering were performed using Fastp, which included automatic adapter detection, poly-G tail trimming (sequencer-specific), and removal of low-quality reads [S3].

Transcript abundance was estimated using Salmon in quasi-mapping-based mode on a decoy-aware gentrome built from Gencode v27 annotations [S4]. Differential gene expression analysis was carried out using the DESeq2 R package (version 1.44.0, R version 4.4.1) [S5]. Genes with an adjusted p-value < 0.05 and an absolute log₂ fold change greater than 1 were considered significantly differentially expressed.

Gene Ontology (GO) enrichment analysis was performed using the clusterProfiler R package [S6]. To account for correlations between multiple samples from the same individual (e.g., paired tumor and normal tissues), we additionally used the Limma + Voom pipeline with the `duplicateCorrelation` function, treating patient ID as a blocking factor.

**Quantitative RT-PCR analysis**

The 2^-ΔΔCq^ method was used to determine the relative abundance of the *IL32*, *BIRC3* and *ACE1* mRNAs in the various types of muscle fragments as well as in cultured myoblasts subjected *in vitro* to various experimental conditions. For muscle fragments, in most cases, we calculated the relative concentration of *IL32*, *BIRC3* and *ACE1* mRNAs, using the 2^-ΔCq^ formula with the *PPIA* (Peptidylprolyl Isomerase A) mRNA as the internal calibrator. In some cases, we calculated the *IL32*/*ACE1* and *BIRC3*/*ACE1* mRNA ratio using the 2^-ΔCq^ formula with the *ACE1* mRNA as the reference. For cultured myoblasts, we used the 2^-^^ΔΔCq^ formula with the *PPIA* mRNA as the internal calibrator and the control condition as the external calibrator, arbitrarily set at one.

**Cell lines propagated *in vitro***

They were grown in Dulbecco’s modified Eagle’s medium (DMEM) (Gibco - ThermoFisher supplemented with 5% fetal calf serum (FCS) (Invitrogen). Pools of normal neonatal human epidermal keratinocytes (NHEK-Neo – ref. 00192906) were purchased from Lonza Bioscience (Basel – Switzerland). They were grown in the culture medium provided by Lonza Bioscience: KGM TM Gold Keratinocyte Growth Medium BulletKitTM (ref. 00192060). In the proliferation setting, they were grown using DMEM mixed with 199 medium (25%); (Merck-Sigma-Aldrich), supplemented with 15% FCS and the following additives: 0.2 µg/ml dexamethasone, 10 ng/ml FGFβ and penicillin/streptomycin (100 µg/ml) instead of gentamycin.

**References**

S3. Chen, S., et al., fastp: an ultra-fast all-in-one FASTQ preprocessor. Bioinformatics, 2018. 34(17): p. i884-i890.

S4. Patro, R., et al., Salmon provides fast and bias-aware quantification of transcript expression. Nat Methods, 2017. 14(4): p. 417-419.

S5. Love, M.I., W. Huber, and S. Anders, Moderated estimation of fold change and dispersion for RNA-seq data with DESeq2. Genome Biol, 2014. 15(12): p. 550.

S6. Yu, G., et al., clusterProfiler: an R package for comparing biological themes among gene clusters. Omics, 2012. 16(5): p. 284-7.
