# Supplementary material for: Enhanced Expression of IL32 mRNA in Skeletal Muscles in the Context of Head and Neck Carcinomas
Source: J Cachexia Sarcopenia Muscle. 2025 Dec 28;17(1):e70160. doi: 10.1002/jcsm.70160 (PMC12745337; doi:10.1002/jcsm.70160)
Supplement: Supplementary file 14 — Data S4: Supplementary Information. [file JCSM-17-e70160-s001.docx]

**Supplementary References**

S1. Ye, Z., et al., Development and Validation of an Automated Image-Based Deep Learning Platform for Sarcopenia Assessment in Head and Neck Cancer. JAMA Netw Open, 2023. 6(8): p. e2328280.

S2. Kubrak, C., et al., Quantifying the severity of sarcopenia in patients with cancer of the head and neck. Clin Nutr, 2024. 43(4): p. 989-1000.

S3. Sun, X.S., et al., Debio 1143 and high-dose cisplatin chemoradiotherapy in high-risk locoregionally advanced squamous cell carcinoma of the head and neck: a double-blind, multicentre, randomised, phase 2 study. Lancet Oncol, 2020. 21(9): p. 1173-1187.
